# Supplementary material for: Contrasting patterns of genome-level diversity across distinct co-occurring bacterial populations
Source: ISME J. 2017 Dec 8;12(3):742–55. doi: 10.1038/s41396-017-0001-0 (PMC5962901; doi:10.1038/s41396-017-0001-0)
Supplement: Supplementary file 2 — Supplemental Text [file 41396_2017_1_MOESM2_ESM.docx]

**Contrasting patterns of genome-level diversity across distinct co-occurring bacterial populations**

Sarahi L Garcia^a,b,*^, Sarah L R Stevens^a,*^, Benjamin Crary^c^, Manuel Martinez-Garcia^d^, Ramunas Stepanauskas^e^, Tanja Woyke^f^, Susannah G Tringe^f^, Siv Andersson^g^, Stefan Bertilsson^b^, Rex R. Malmstrom^f^, Katherine D McMahon^a,c^

a Department of Bacteriology, University of Wisconsin-Madison Madison, WI, USA

b Department of Ecology and Genetics, Limnology and Science for Life Laboratory, Uppsala University, Uppsala, Sweden

c Department of Civil and Environmental Engineering, University of Wisconsin-Madison, Madison, WI, USA

d Department of Physiology, Genetics and Microbiology, University of Alicante, Alicante, Spain.

e Bigelow Laboratory for Ocean Sciences, East Boothbay, ME, USA

f DOE Joint Genome Institute, Walnut Creek, CA, USA

g Department of Molecular Evolution, Uppsala University, S-752 36 Uppsala, Sweden

* Equal contributors

**Supplementary Figures**

**Figure S1.**

Nucleotide identity density plots for SAG versus SAG genome-wide comparison using a sliding window. This is an expanded version of the data presented in Figure 2 of the main text. Results are shown for two reference SAGs representing the most complete genomes from the most thoroughly sampled tribes. All SAG pairs were from the same tribe. Nucleotide identity was calculated with blastn using 301 bp fragments that overlapped by 150 bp.

**Figure S2.**

Mapping metagenomic reads from Lake Mendota to SAGs. One recruitment plot for each SAG showing the identity and location on the genome for each read recruited. The metagenome sample was collected from Lake Mendota on 29 April 2009. Reads were only counted if they aligned over a minimum of 200 bp. Recruitments were not competitive, meaning that each read could recruit to multiple SAGs. Reads were only included if they aligned over a minimum of 200 bp. AAA041L13 had no hits and was not included.

**Figure S3.**

Mapping metagenomic reads from Lake Mendota to SAGs and the composite genomes from (Kang et al 2017). This figure is an expanded version of Figure 3 presented in the main text. The x-axis represents nucleotide identity of the recruited reads. The metagenome sample was collected from Lake Mendota on 29 April 2009. Reads were only counted if they aligned over a minimum of 200 bp. Recruitments were not competitive, meaning that each read could recruit to multiple SAGs. Color coding matches figures in the main text.

**Figure S4.**

Mapping metagenomic reads from Lake Mendota to SAGs. The x-axis represents nucleotide identity of the recruited reads. The metagenome sample was collected from Lake Mendota on 29 April 2009. Reads were only counted if they aligned over a minimum of 200 bp. Recruitment was performed competitively, meaning that each query was only counted for the SAG or SAGs to which it hit best.

**Figure S5.**

Total acI and LD12 population abundance in Lake Mendota, as measured by the relative number of reads recruited to each SAG using blastn. All SAGs and samples are from Lake Mendota. Timepoints are pooled by month. Filtering criteria: ≥97.5% ANI and ≥200 bp alignment length. Blastn was done competitively, only counting the read for the best hit genome with the exception of when it hit equally well, then counted for all best hit genomes. Values are expressed as the sum of reads recruited for each group (not relativized since these were pooled for a each group and could not be normalized by genome size).

**Figure S6.**

Seasonal patterns of abundance for LD12 sequence-discrete populations from Lake Mendota represented by five different SAGs. Filtering criteria: ≥97.5% ANI and ≥200 bp alignment length. Relative abundance represents the average coverage per base in the reference SAG divided by the size of the metagenome, multiplied by the average size of all metagenomes (1.34 Gigabases). Note that the resulting values do not represent a true measure of absolute abundance, but allow for quantitative comparison of month-to-month variation in population-level abundance. Dark line shows the median, and boundaries of the box are third and first quartile. Whiskers show maximum and minimum, and circles show outliers.

**Figure S7.**

Seasonal patterns of abundance for acI sequence-discrete populations from Lake Mendota represented by three different SAGs. Filtering criteria: ≥97.5% ANI and ≥200 bp alignment length. Relative abundance represents the average coverage per base in the reference SAG divided by the size of the metagenome, multiplied by the average size of all metagenomes (1.34 Gigabases). Note that the resulting values do not represent a true measure of absolute abundance, but allow for quantitative comparison of month-to-month variation in population-level abundance. Dark line shows the median, and boundaries of the box are third and first quartile. Whiskers show maximum and minimum, and circles show outliers.

**Figure S8.**

Heat map displaying correlations between the relative abundance of populations within the LD12 and acI-tribes. Relative abundance was calculated using recruited metagenomic reads, as follows. Metagenomic reads from Lake Mendota were recruited against each SAG using blastn with the following filtering criteria: ≥97.5% ANI and ≥200 bp alignment length. Metagenomes were pooled by month. Normalized coverage was calculated as the average coverage per base in the reference SAG divided by the size of the metagenome pool. Relative abundance was calculated by multiplying the normalized coverage by the average size of all metagenome pools (3.5 Gigabases). Correlations were calculated using the non-parametric Spearman rank correlation. Data used for the correlations are provided in Table S5.

**Tables**

**Table S1.** Classification of the nine SAGs that were not members of the acI lineage or LD12 tribe, based on PhyloSift (Darling et al 2014).

**Table S2.** Comparisons of all SAG pairs. The 16S rRNA gene alignment length and identity were calculated using the longest available sequences, either from the SAG assembly or from the PCR products originally used to screen the SAG libraries (Martinez-Garcia et al 2011). The gANI and alignment fraction (AF) were calculated using ANIcalculator (Varghese et al 2015).

**Table S3.** An estimate of the extent to which the SAGs represent the complete population-level diversity of each lineage present in the lake. The number of reads that were recruited at above 97.5% identity was used as a measure of the abundance of the population represented by that SAG. The number of reads that recruited above 60% identity was used as a measure of the abundance of the lineage represented by that SAG. Thus, the first number divided by the second number should yield 100% if we have adequately sampled all of the populations within the lineage, using our SAG collection.

**Table S4.** Metadata for the metagenomic time series. Includes the GOLD project ID, sequencing library code, number of reads in library that merged and passed quality filtering, number of base pairs in library that merged and passed quality filtering, layer of the lake the sample came from, date sample was collected, and JGI Portal ID.

**Table S5.** Correlations between the relative abundance of each population. Relative abundance was calculated using recruited metagenomic reads, as follows. Metagenomic reads from Lake Mendota were recruited against each SAG using blastn with the following filtering criteria: ≥97.5% ANI and ≥200 bp alignment length. Metagenomes were pooled by month. Normalized coverage was calculated as the average coverage per base in the reference SAG divided by the size of the metagenome pool. Relative abundance was calculated by multiplying the normalized coverage by the average size of all metagenome pools (3.5 Gigabases). Data used for the correlations are provided in Table S6 except in the case of LD12 SAGs and acI-B1 SAGs, which were subjected to a more strict criterion for counting reads in a competitive way. That is, if a read matched more than one SAG equally well, it was not counted at all. Thus, the number of reads recruited per SAG was in some cases lower, but usually only minimally so.

**Table S6.** Recruitment results for data presented in Figure 5. Metagenomic reads from Lake Mendota were recruited against each SAG using blastn with the following filtering criteria: ≥97.5% ANI and ≥200 bp alignment length. Metagenomes were pooled by month. Raw hit counts per SAG are provided. Normalized coverage was calculated as the average coverage per base in the reference SAG divided by the size of the metagenome pool. Relative abundance was calculated by multiplying the normalized coverage by the average size of all metagenome pools (3.5 Gigabases). Note that the resulting values do not represent a true measure of absolute abundance, but allow for quantitative comparison of month-to-month variation in population-level abundance.

**References**

Darling AE, Jospin G, Lowe E, Matsen FAt, Bik HM, Eisen JA (2014). PhyloSift: phylogenetic analysis of genomes and metagenomes. PeerJ 2: e243.

Kang I, Kim S, Islam MR, Cho JC (2017). The first complete genome sequences of the acI lineage, the most abundant freshwater Actinobacteria, obtained by whole-genome-amplification of dilution-to-extinction cultures. Sci Rep 7: 42252.

Martinez-Garcia M, Swan BK, Poulton NJ, Gomez ML, Masland D, Sieracki ME *et al* (2011). High-throughput single-cell sequencing identifies photoheterotrophs and chemoautotrophs in freshwater bacterioplankton. ISME J.

Varghese NJ, Mukherjee S, Ivanova N, Konstantinidis KT, Mavrommatis K, Kyrpides NC *et al* (2015). Microbial species delineation using whole genome sequences. Nucleic Acids Res 43: 6761-6771.
