# Supplementary material for: Contrasting patterns of genome-level diversity across distinct co-occurring bacterial populations
Source: ISME J. 2017 Dec 8;12(3):742–55. doi: 10.1038/s41396-017-0001-0 (PMC5962901; doi:10.1038/s41396-017-0001-0)
Supplement: Supplementary file 3 — Supplemental Figures [file 41396_2017_1_MOESM3_ESM.zip › FigS2-dotplots-toc-20160808.pdf]

Figure S1 Recruitment plots

Table of contents

1. Table of contents
2. AAA023D18
3. AAA023J06
4. AAA023L09
5. AAA024D14
6. AAA024N17
7. AAA027C02
8. AAA027C06
9. AAA027D23
10. AAA027G08
11. AAA027I06
12. AAA027I19
13. AAA027J10
14. AAA027J17
15. AAA027K21
16. AAA027L06
17. AAA027L15
18. AAA027M14
19. AAA027N21
20. AAA028A23
21. AAA028C07
22. AAA028D10
23. AAA028I14
24. AAA028K02
25. AAA028N15
26. AAA044D11
27. AAA044N04
28. AAA278I18
29. AAA278O22
30. AAA280B11
31. AAA280P20
32. AAA487M09
33. AB141P03

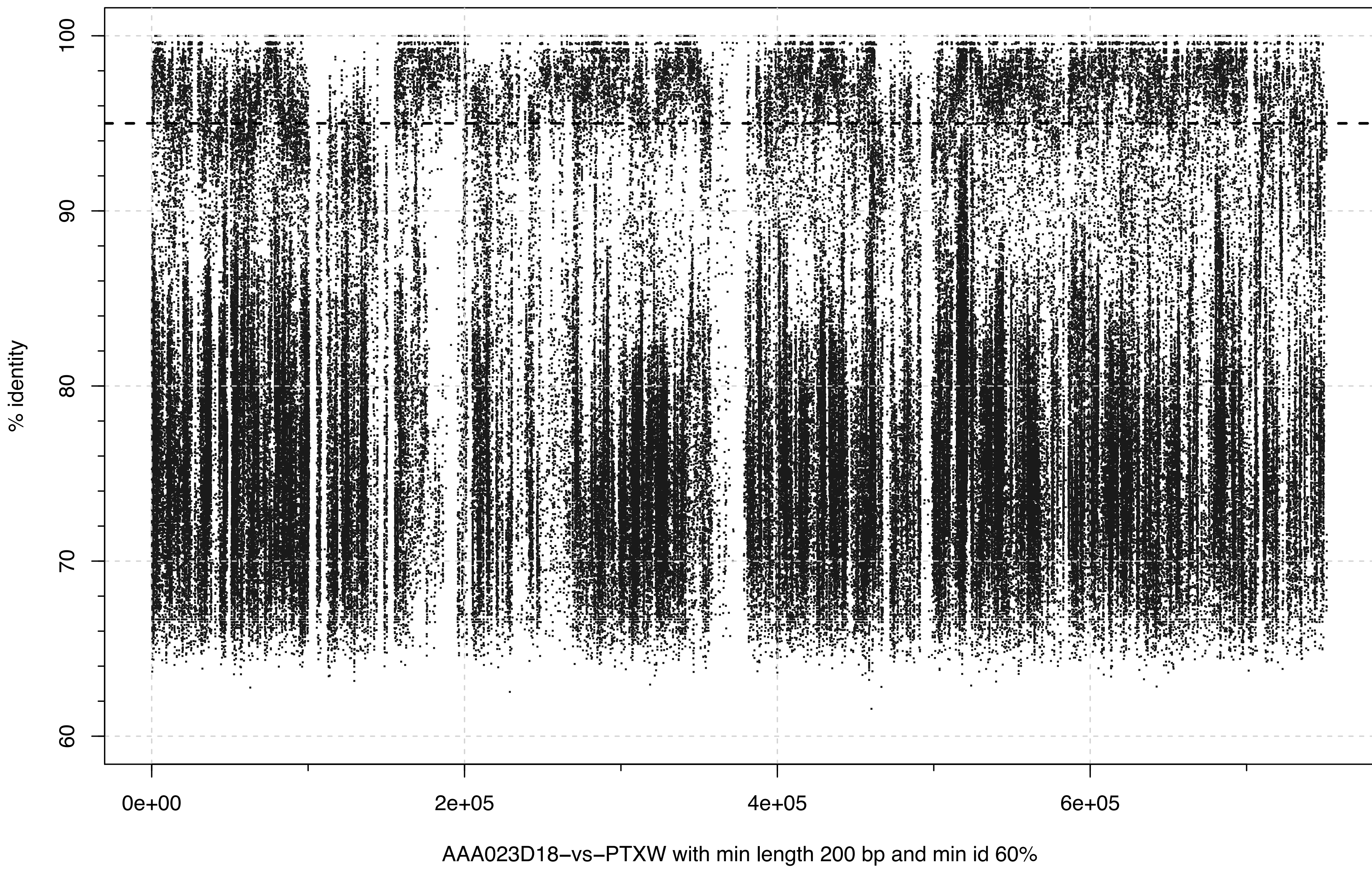

% identity
100
90
80
70
60
0e+00
2e+05
4e+05
6e+05
AAA023D18−vs−PTXW with min length 200 bp and min id 60%

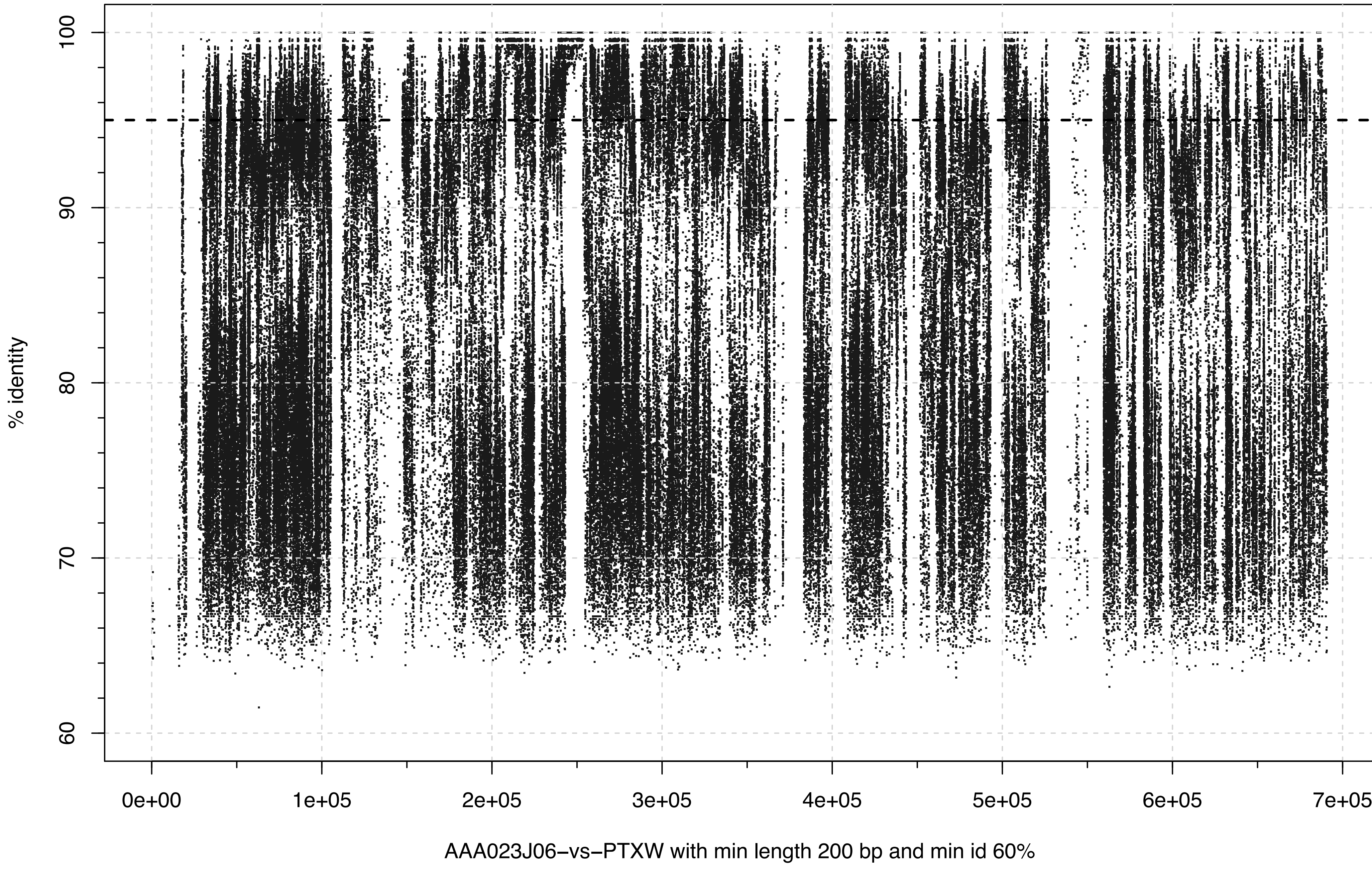

100
90
80
70
60
% identity
0e+00
1e+05
2e+05
3e+05
4e+05
5e+05
6e+05
7e+05
AAA023J06−vs−PTXW with min length 200 bp and min id 60%

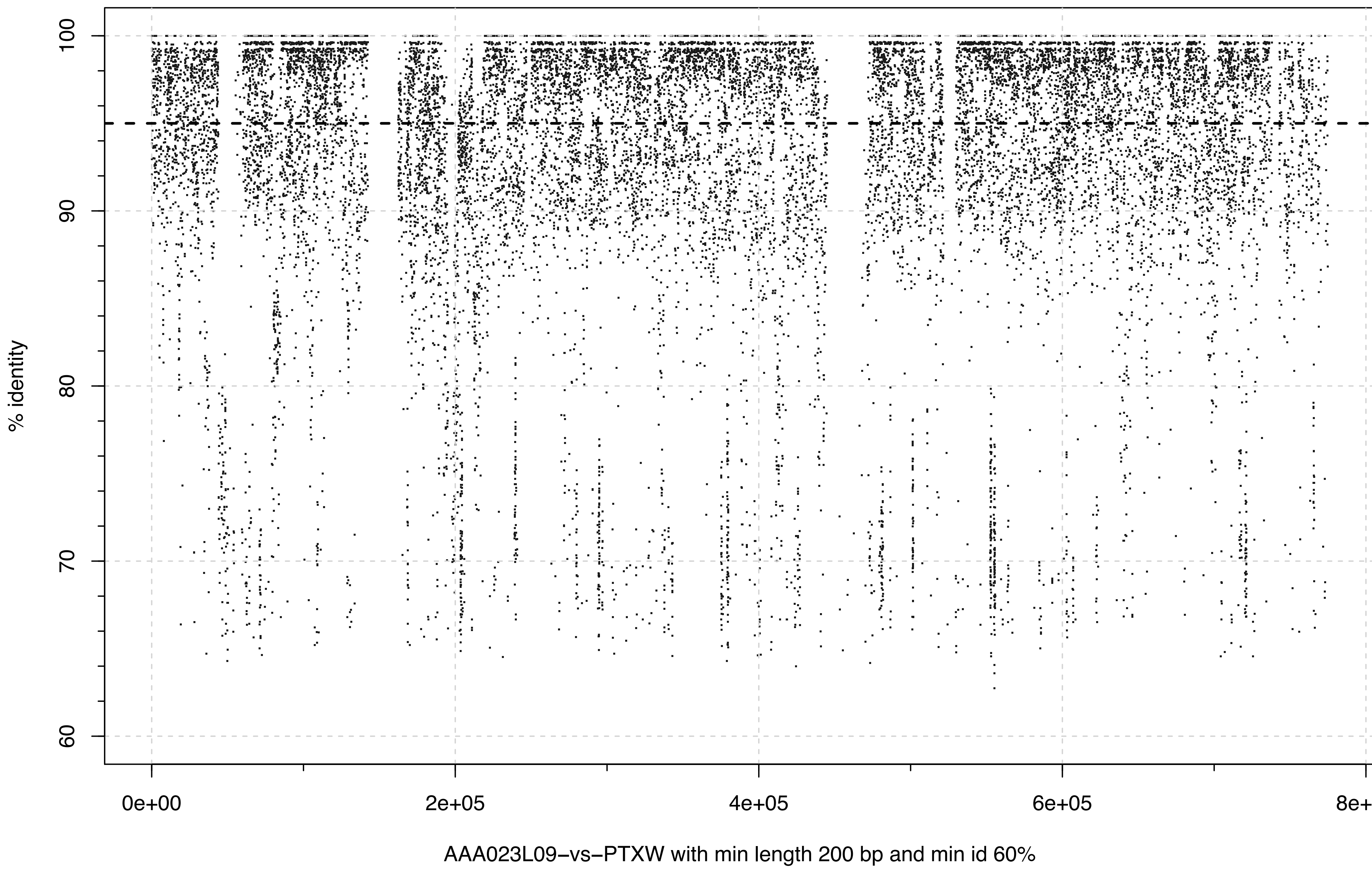

100
90
80
70
60
% identity
0e+00
2e+05
4e+05
6e+05
8e+
AAA023L09−vs−PTXW with min length 200 bp and min id 60%

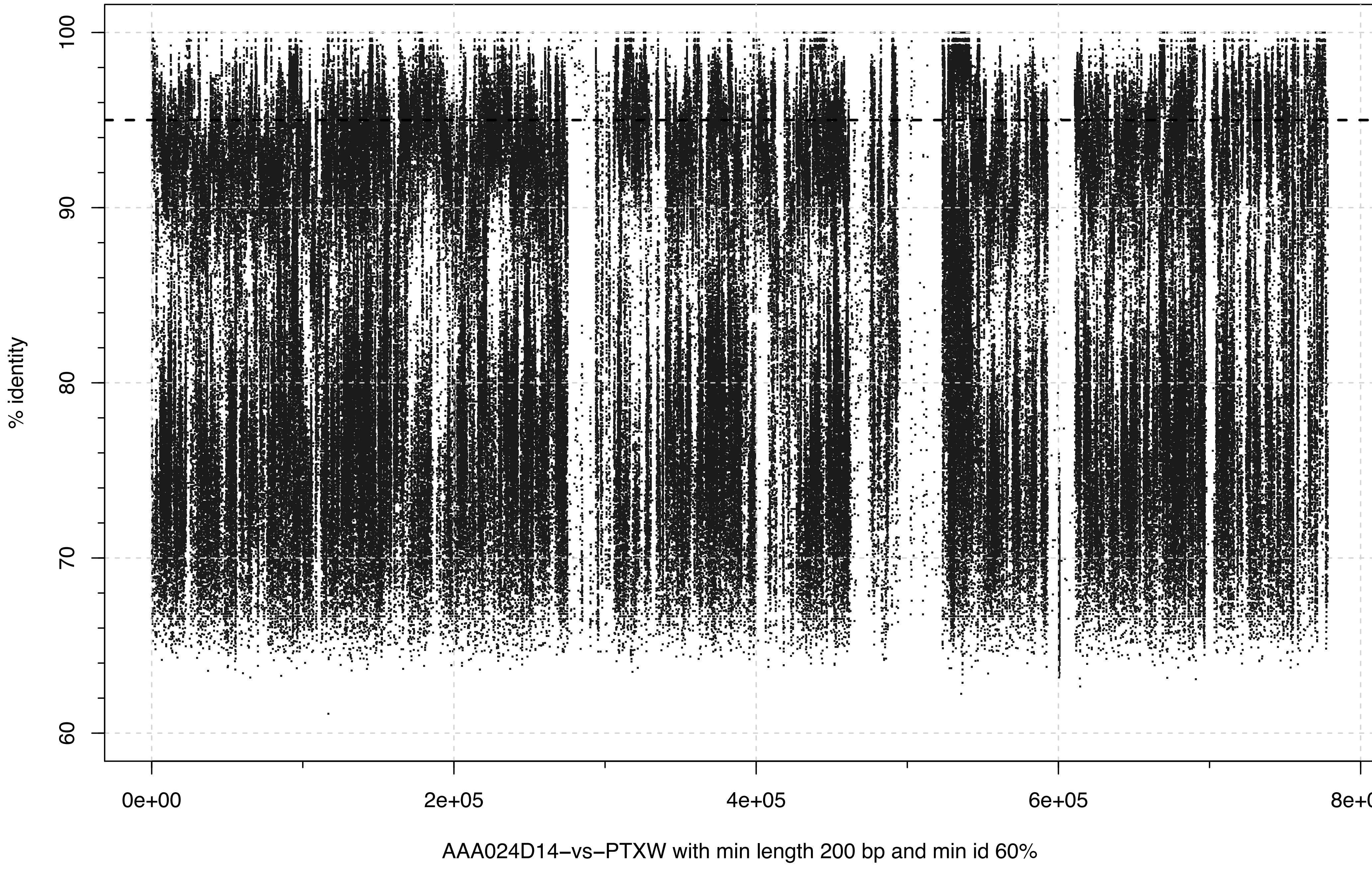

100
90
80
70
60
% identity
0e+00
2e+05
4e+05
6e+05
8e+0
AAA024D14−vs−PTXW with min length 200 bp and min id 60%

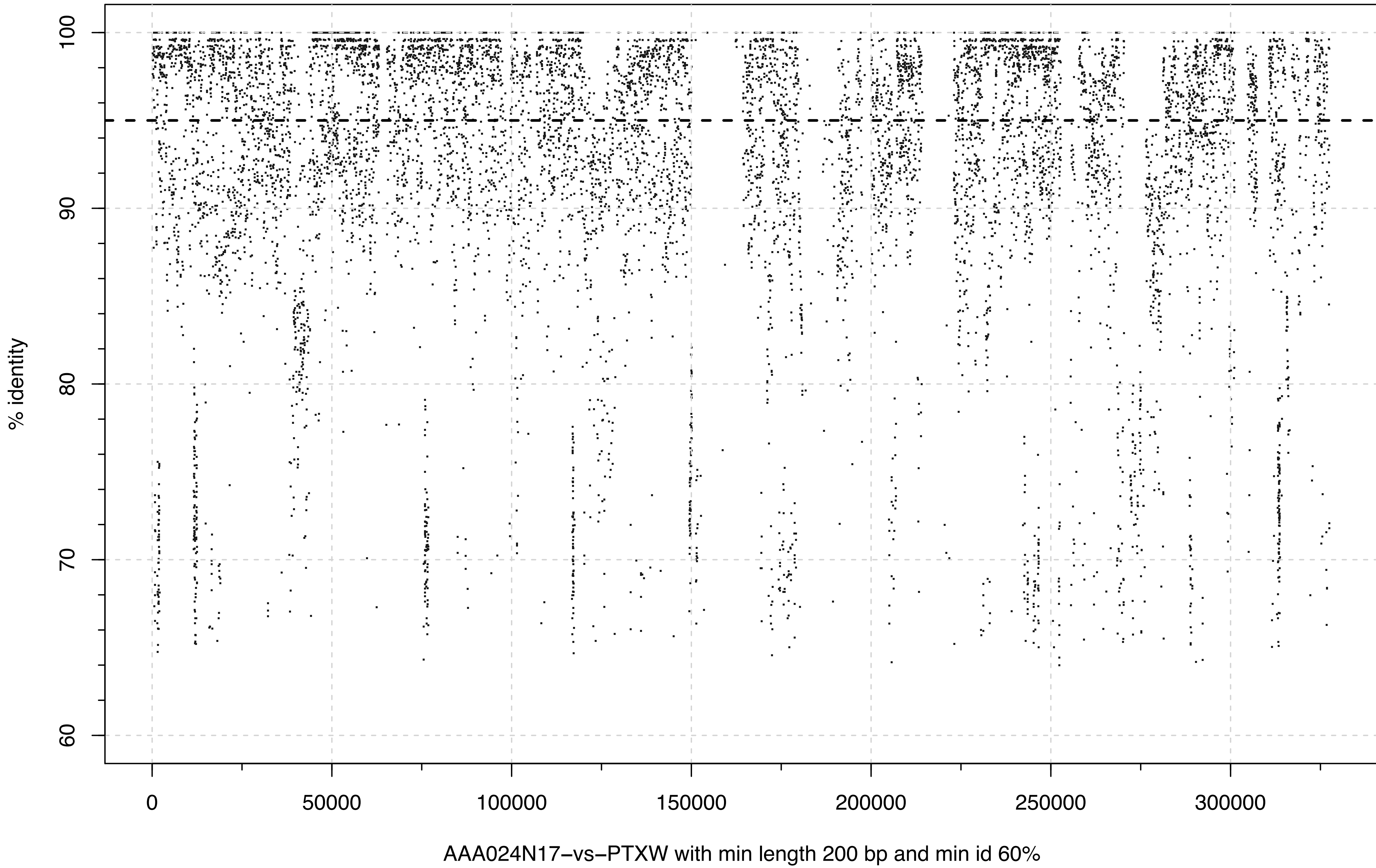
% identity
100
90
80
70
60
0
50000
100000
150000
200000
250000
300000
AAA024N17–vs–PTXW with min length 200 bp and min id 60%

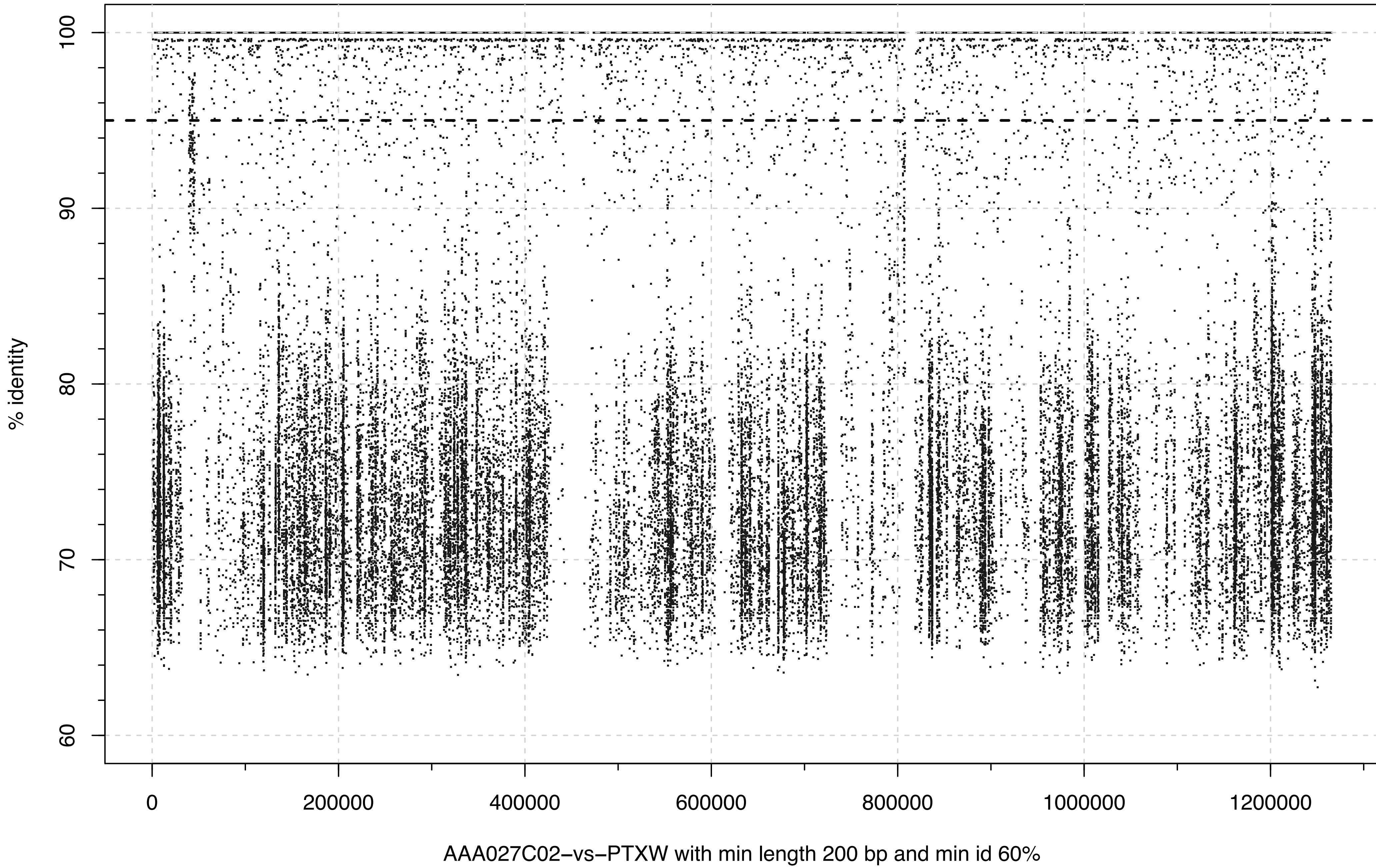

100
90
80
70
60
% identity
0
200000
400000
600000
800000
1000000
1200000
AAA027C02−vs−PTXW with min length 200 bp and min id 60%

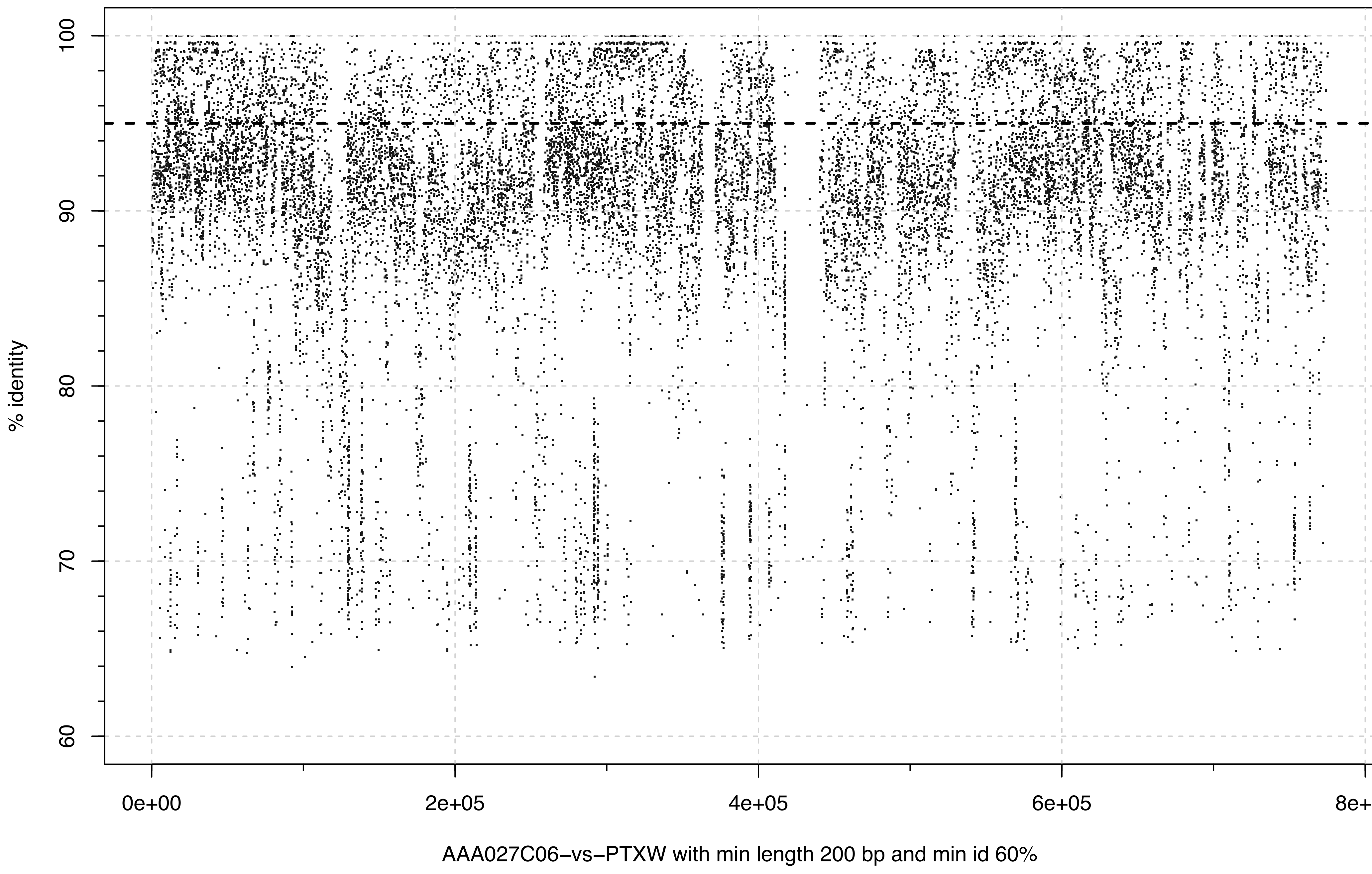

% identity
100
90
80
70
60
0e+00
2e+05
4e+05
6e+05
8e+
AAA027C06−vs−PTXW with min length 200 bp and min id 60%

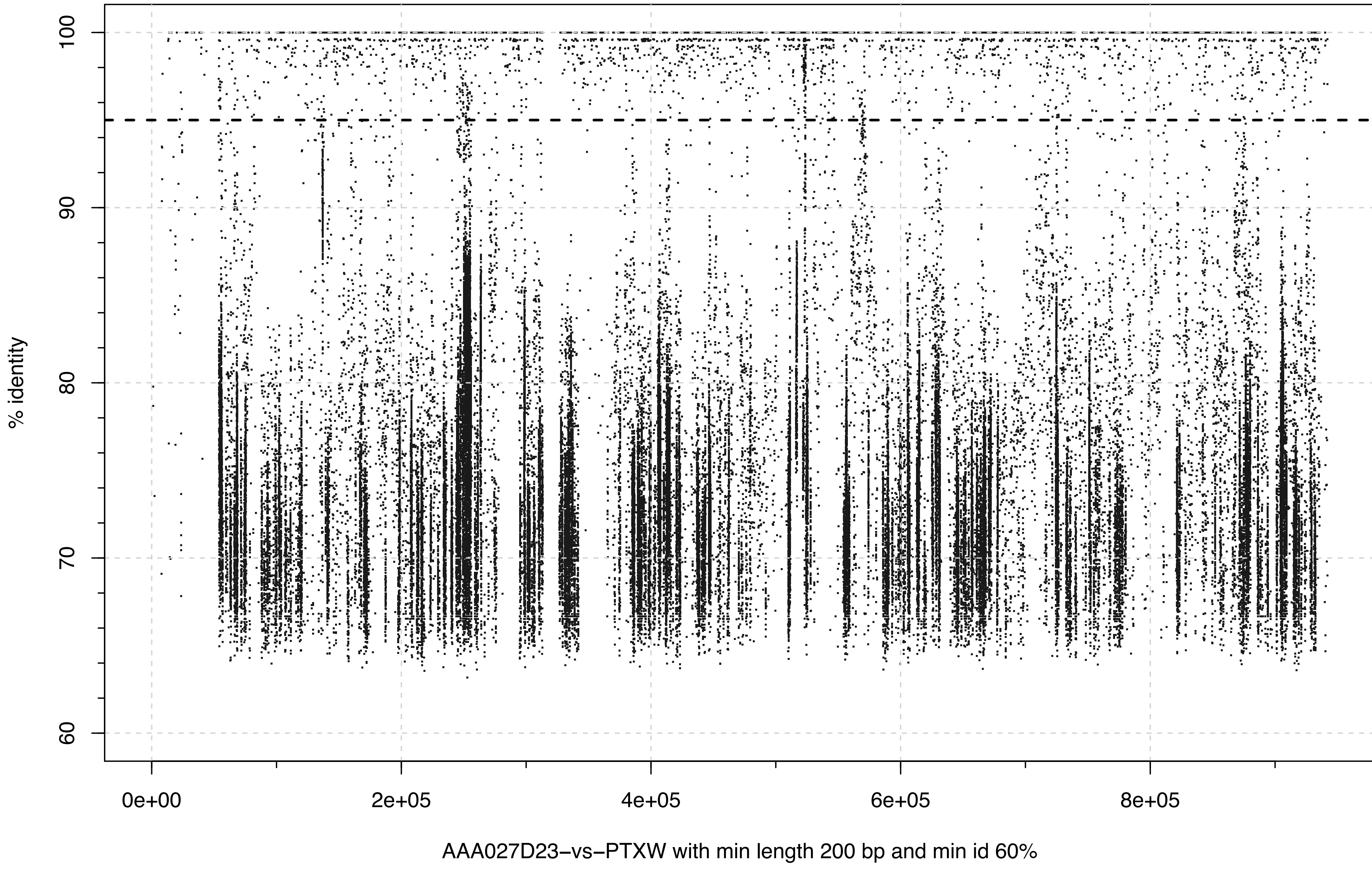

100
90
80
70
60
% identity
0e+00
2e+05
4e+05
6e+05
8e+05
AAA027D23−vs−PTXW with min length 200 bp and min id 60%

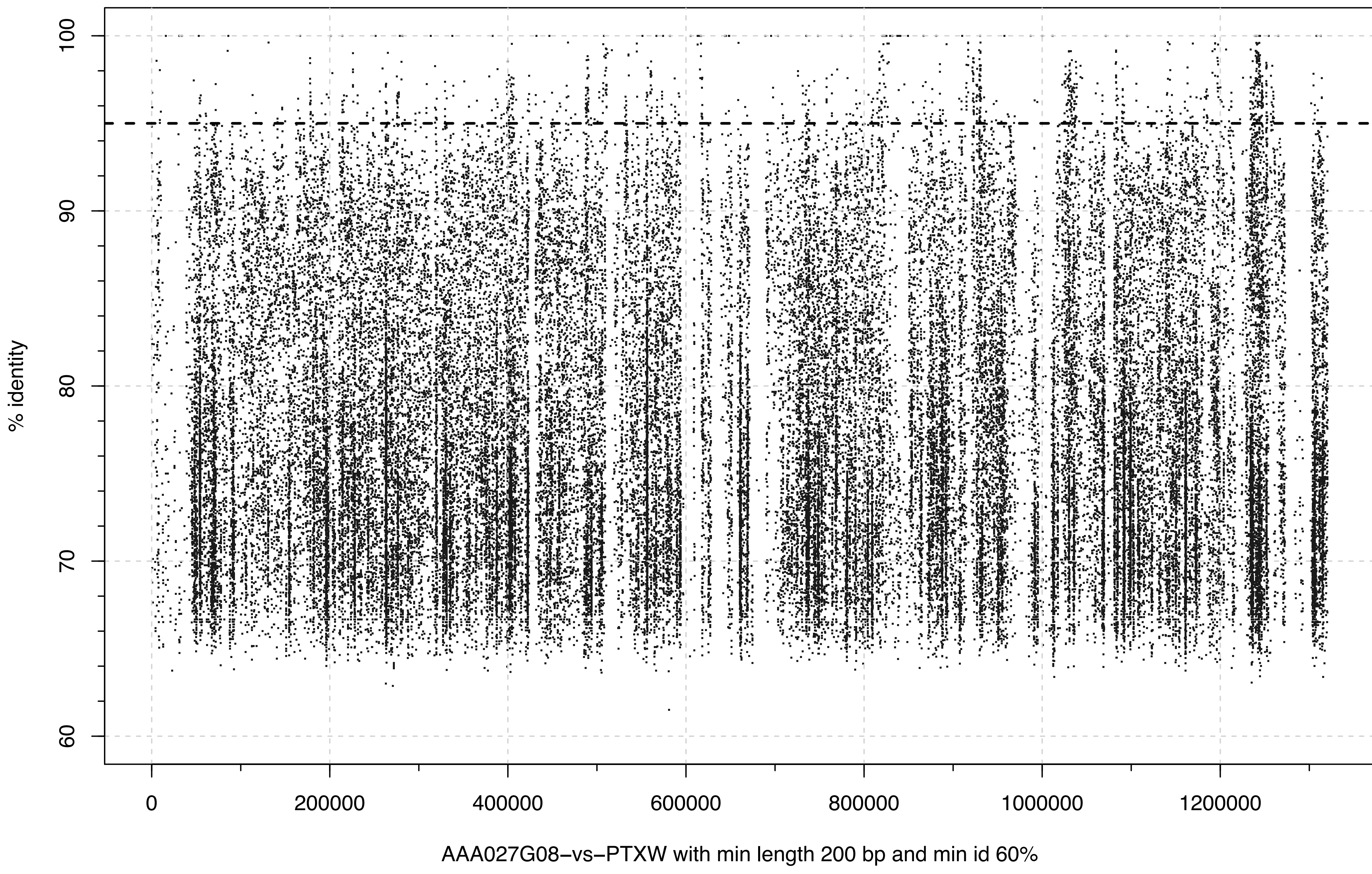

% identity
100
90
80
70
60
0
200000
400000
600000
800000
1000000
1200000
AAA027G08−vs−PTXW with min length 200 bp and min id 60%

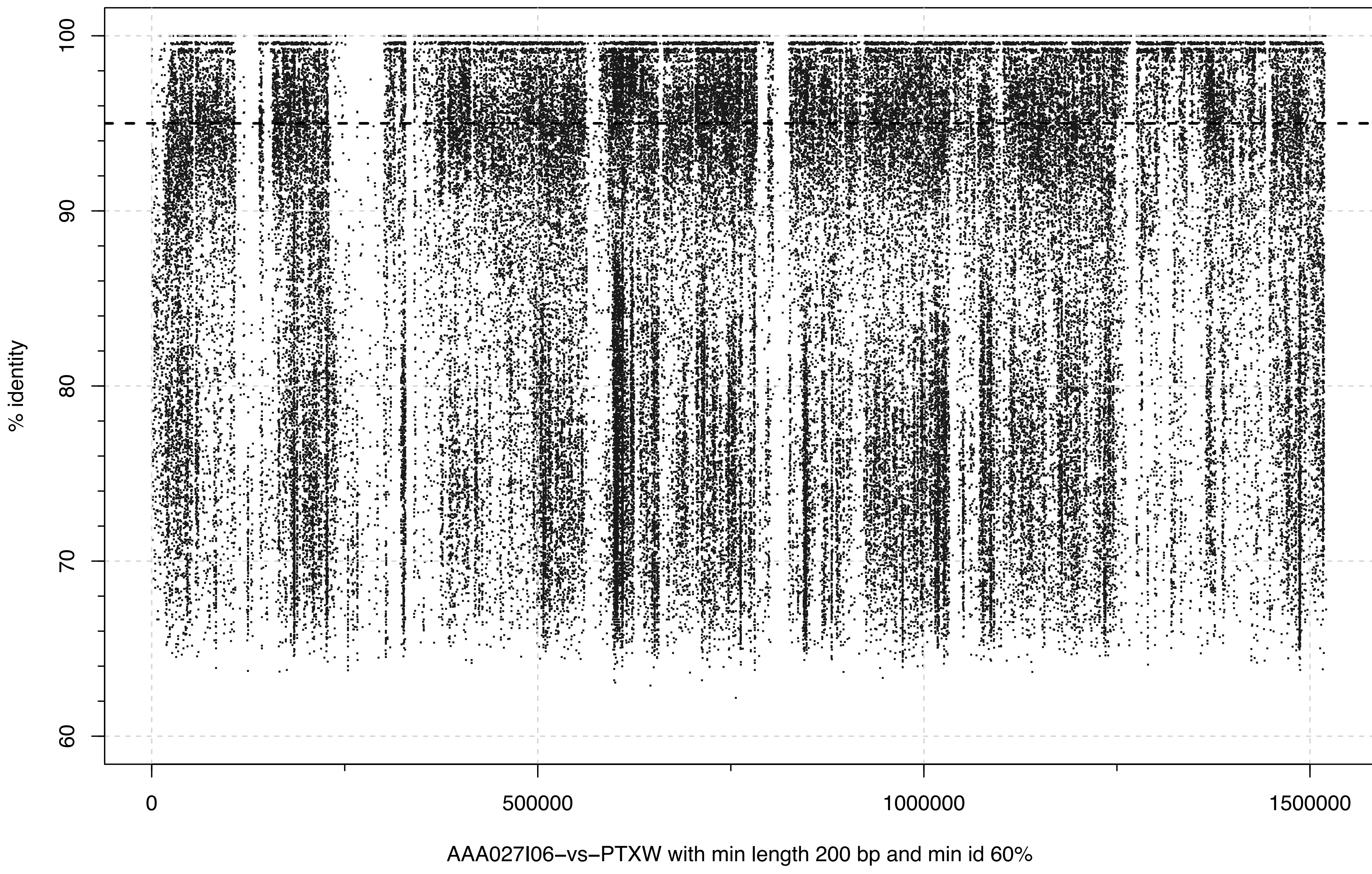

% identity
100
90
80
70
60
0
500000
1000000
1500000
AAA027I06–vs–PTXW with min length 200 bp and min id 60%

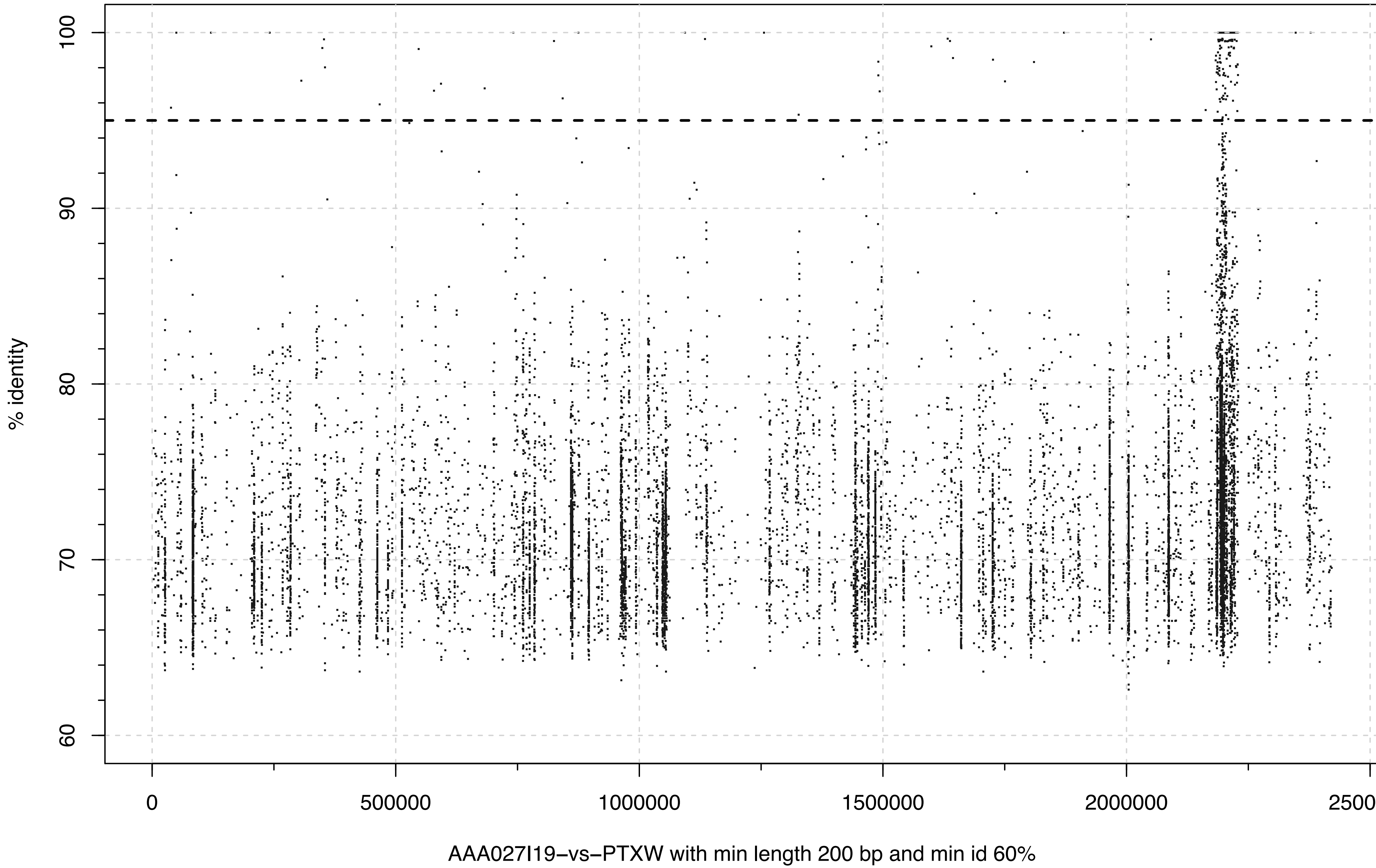

100
90
80
70
60
% identity
0
500000
1000000
1500000
2000000
AAA027I19−vs−PTXW with min length 200 bp and min id 60%

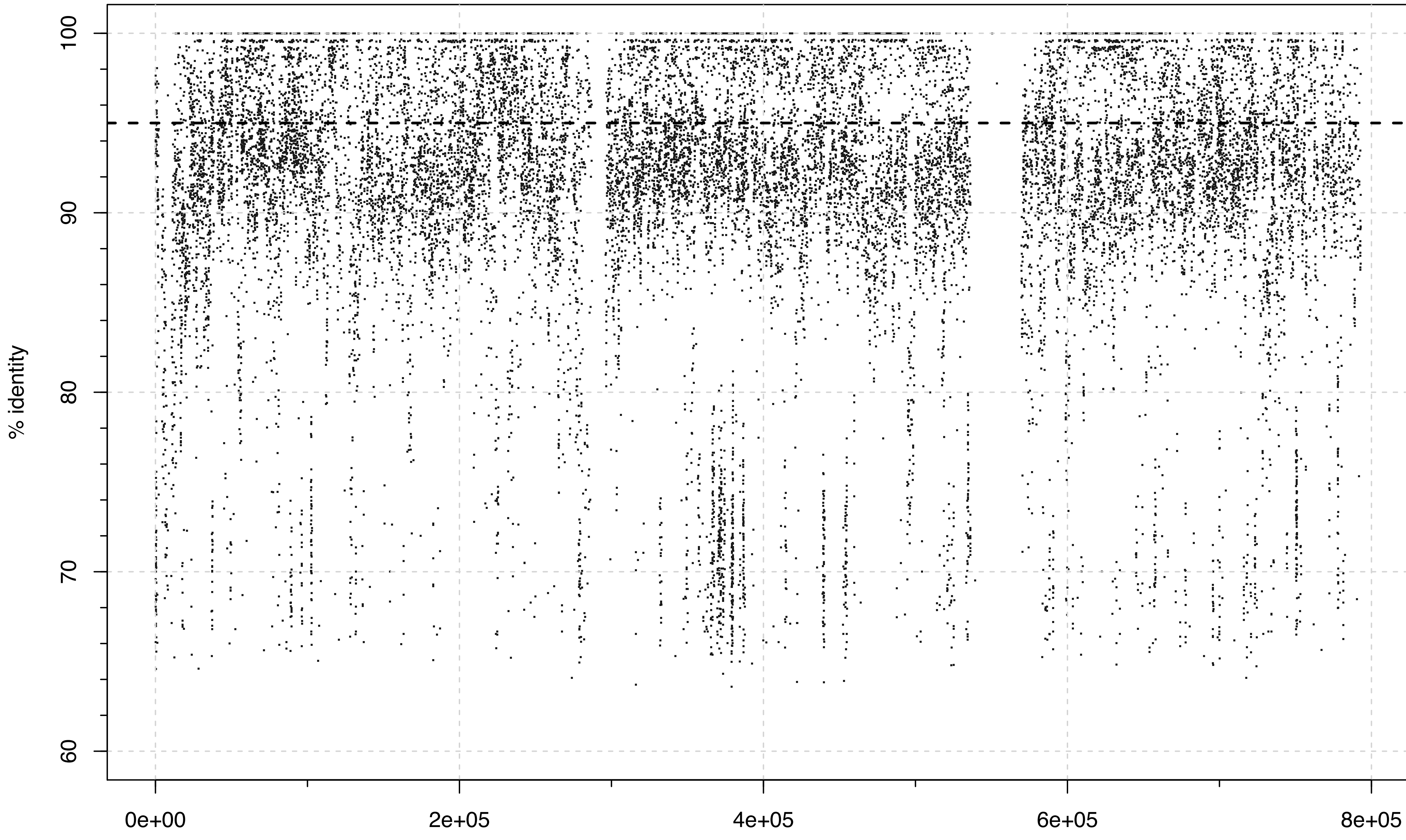

AAA027J10−vs−PTXW with min length 200 bp and min id 60%

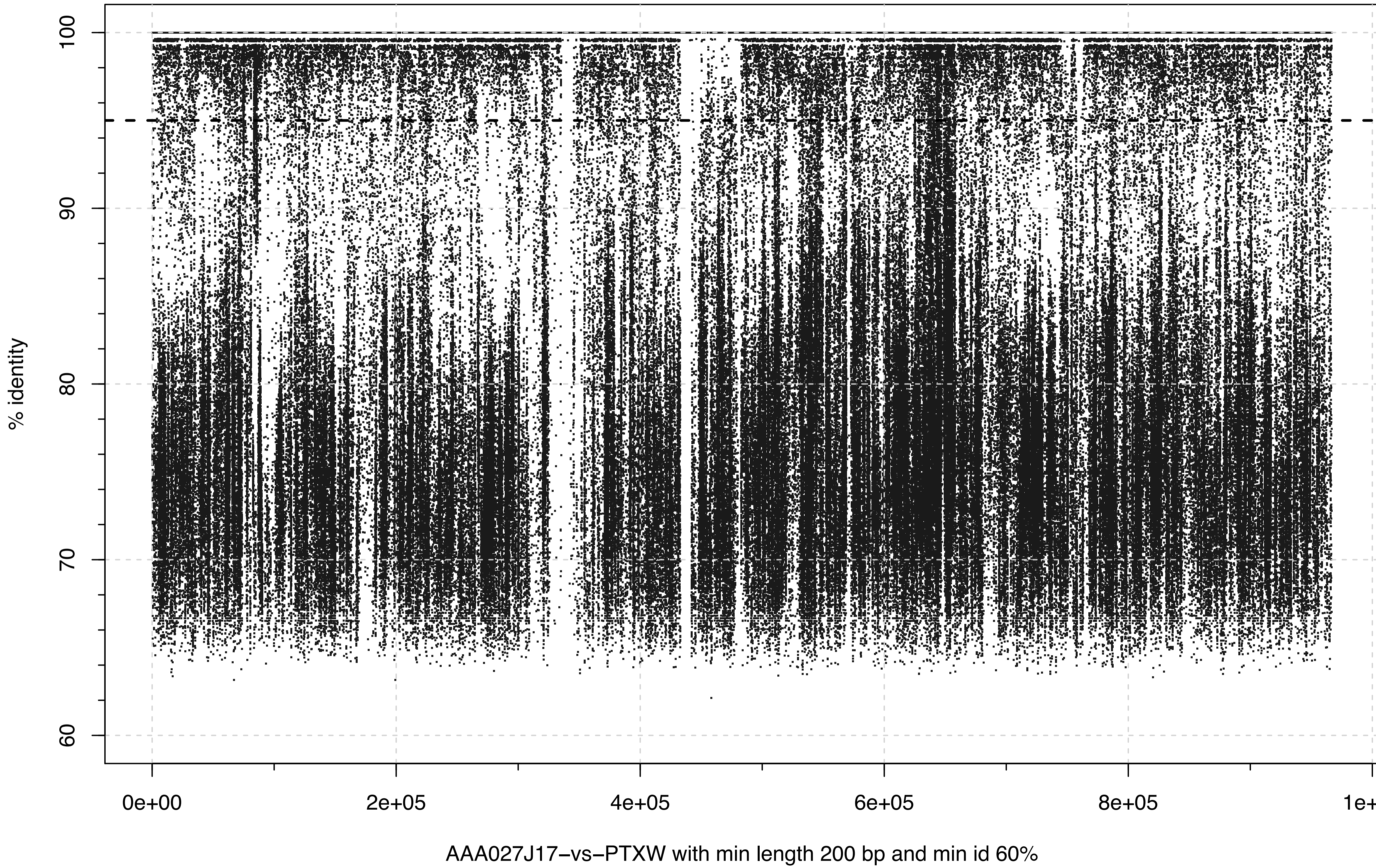

100
90
80
70
60
% identity
0e+00
2e+05
4e+05
6e+05
8e+05
1e+
AAA027J17−vs−PTXW with min length 200 bp and min id 60%

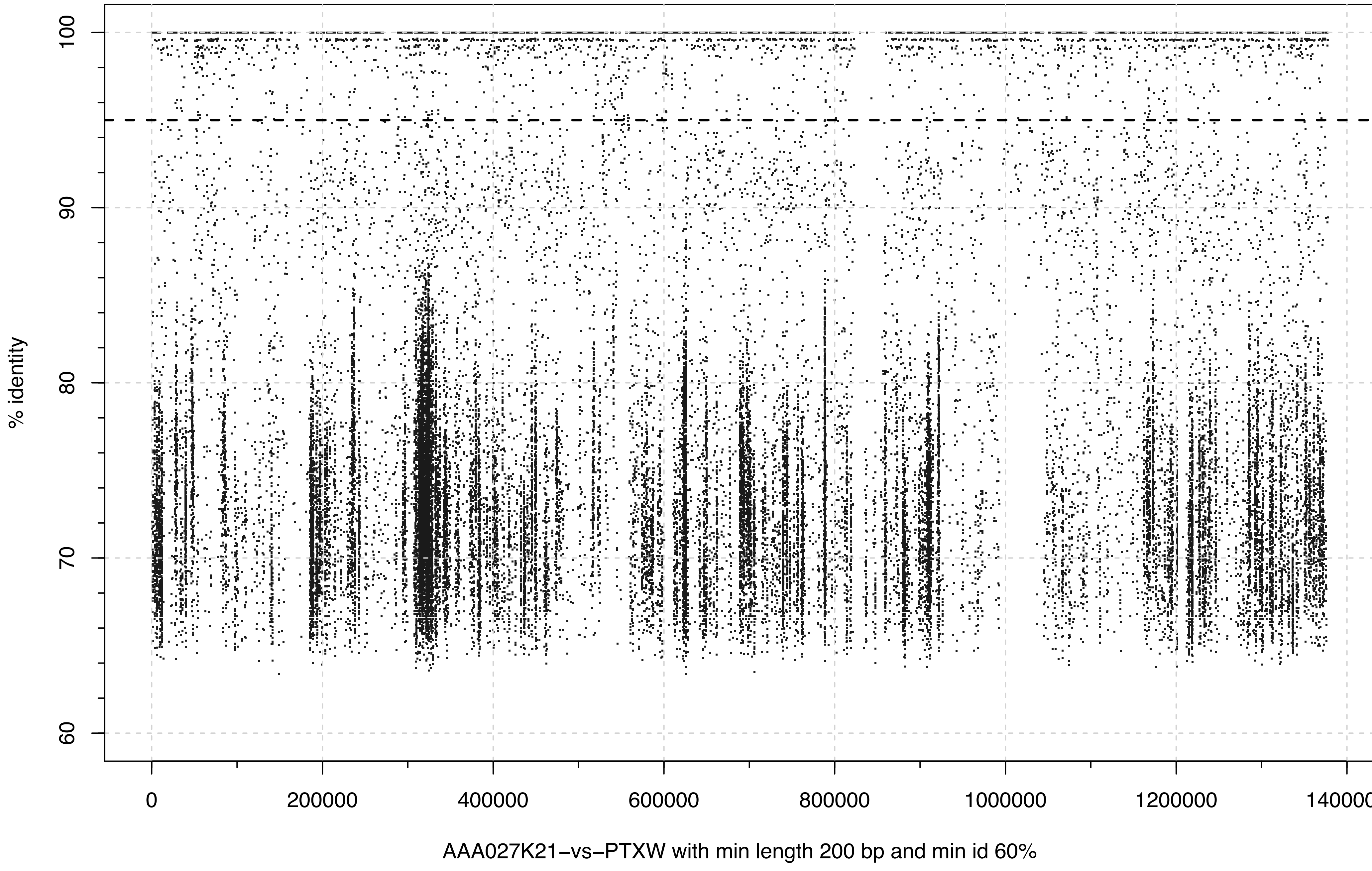

100
90
80
70
60
% identity
0
200000
400000
600000
800000
1000000
1200000
140000
AAA027K21−vs−PTXW with min length 200 bp and min id 60%

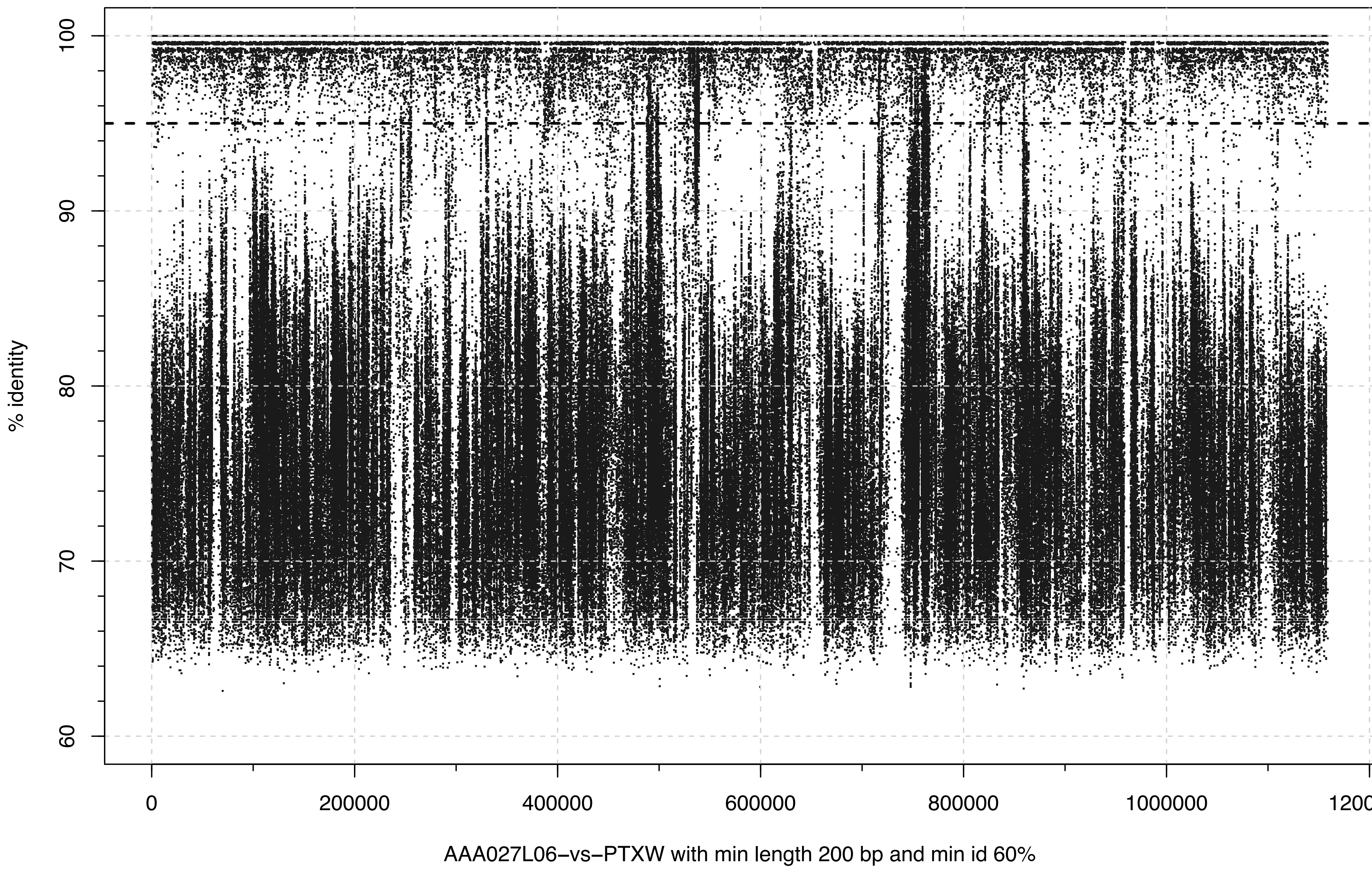

% identity
100
90
80
70
60
0
200000
400000
600000
800000
1000000
1200
AAA027L06–vs–PTXW with min length 200 bp and min id 60%

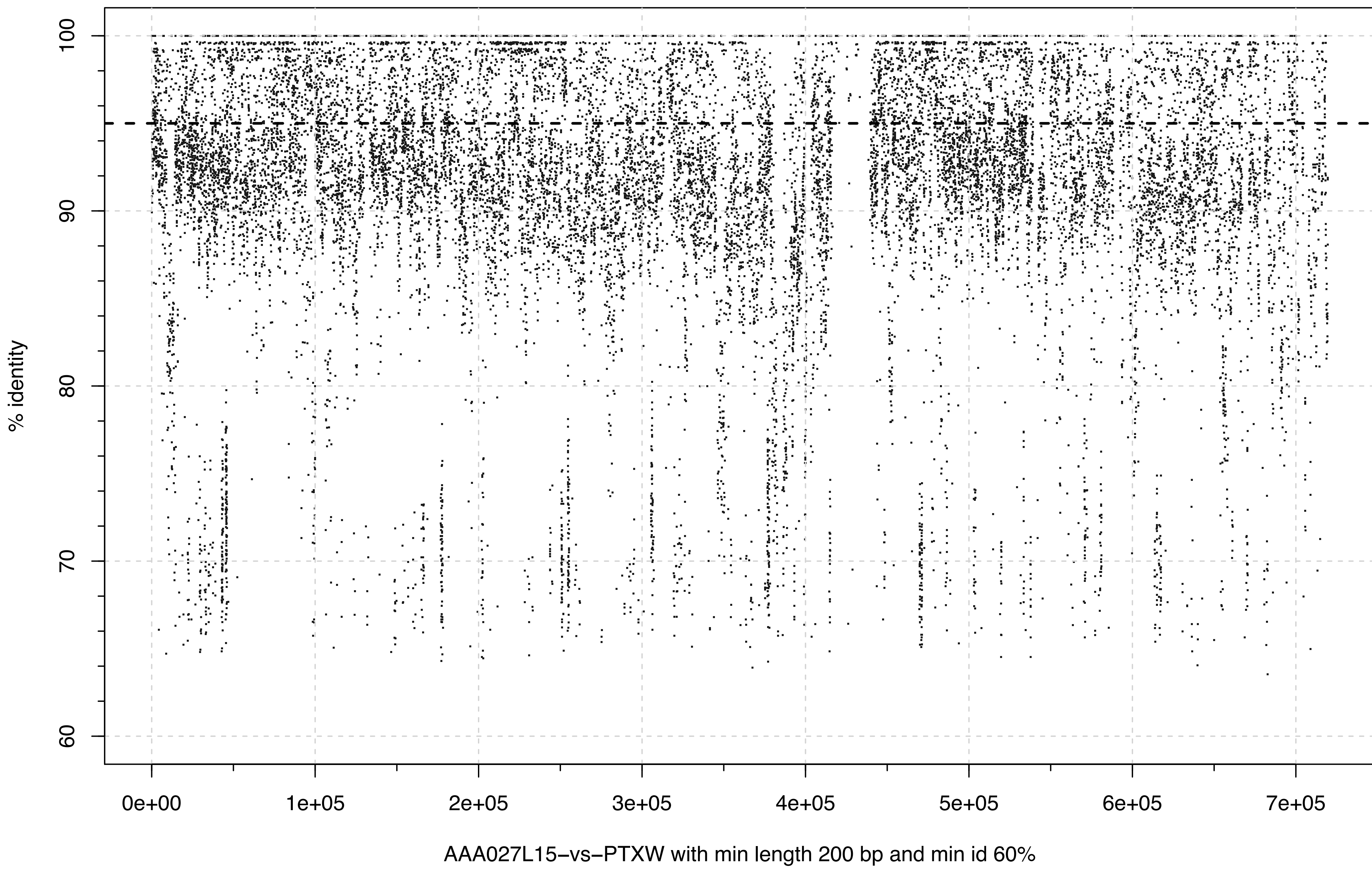
100
90
80
70
60
% identity
0e+00
1e+05
2e+05
3e+05
4e+05
5e+05
6e+05
7e+05
AAA027L15−vs−PTXW with min length 200 bp and min id 60%

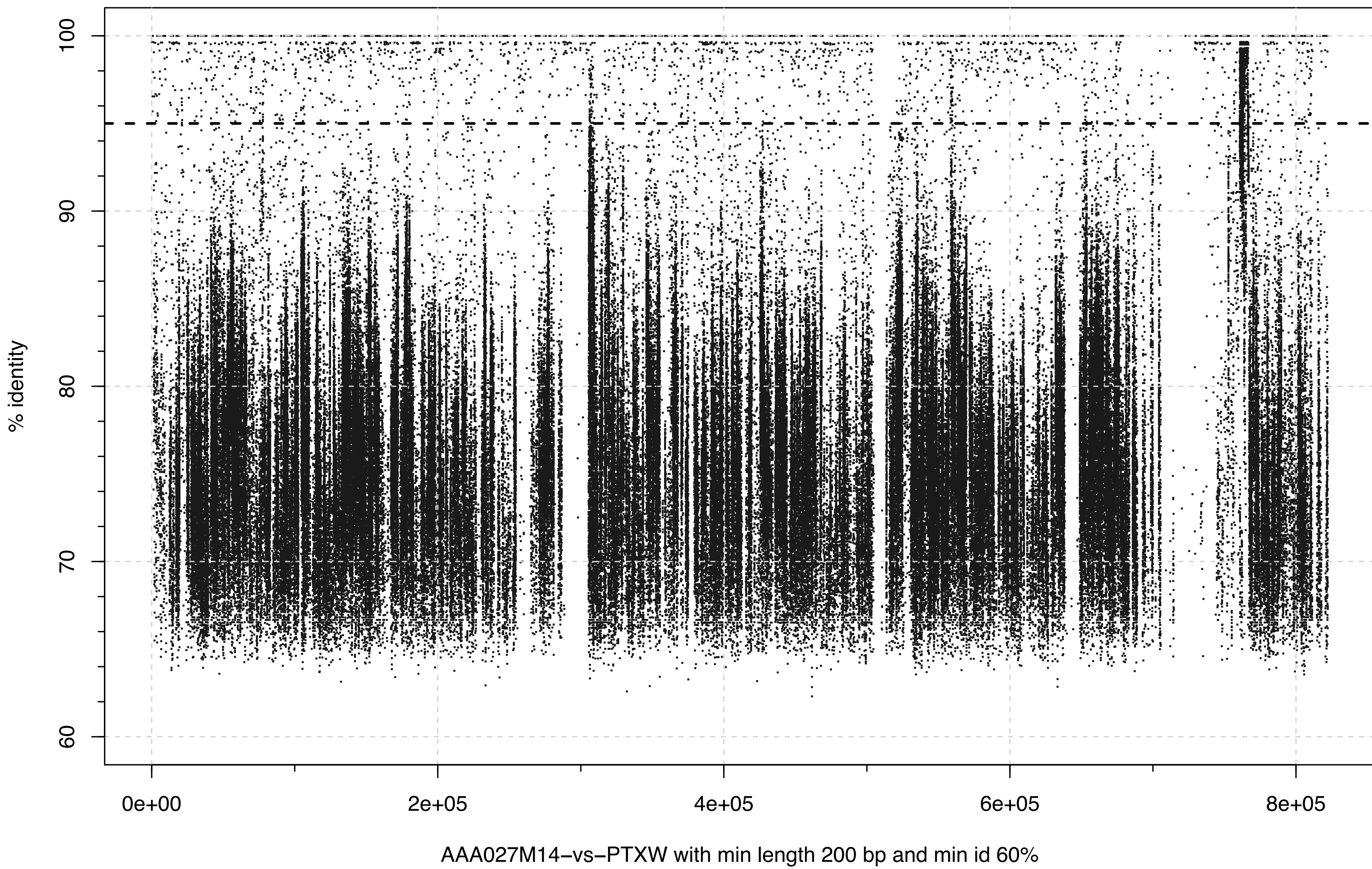

100
90
80
70
60
% identity
0e+00
2e+05
4e+05
6e+05
8e+05
AAA027M14−vs−PTXW with min length 200 bp and min id 60%

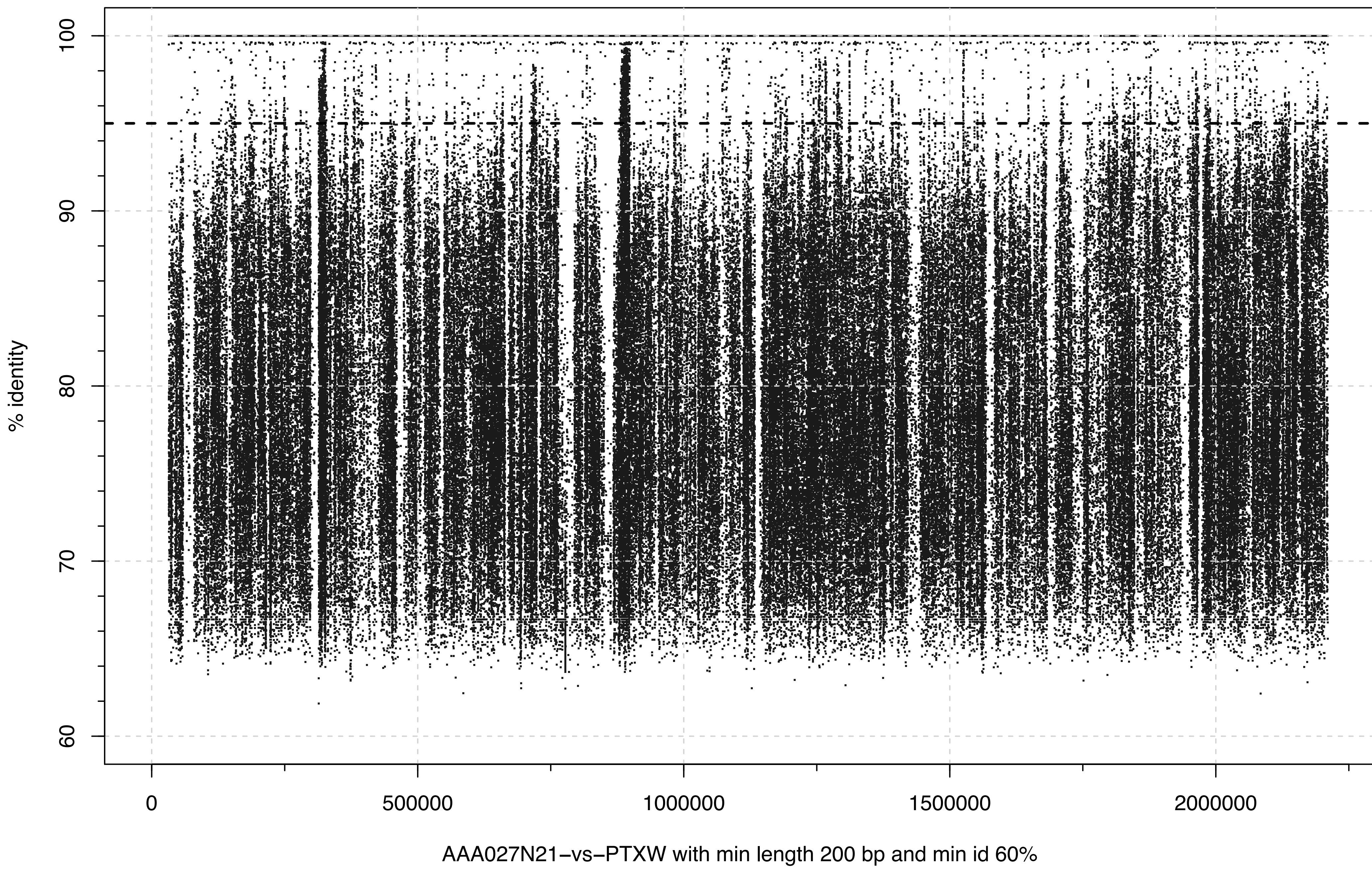

% identity
100
90
80
70
60
0
500000
1000000
1500000
2000000
AAA027N21−vs−PTXW with min length 200 bp and min id 60%

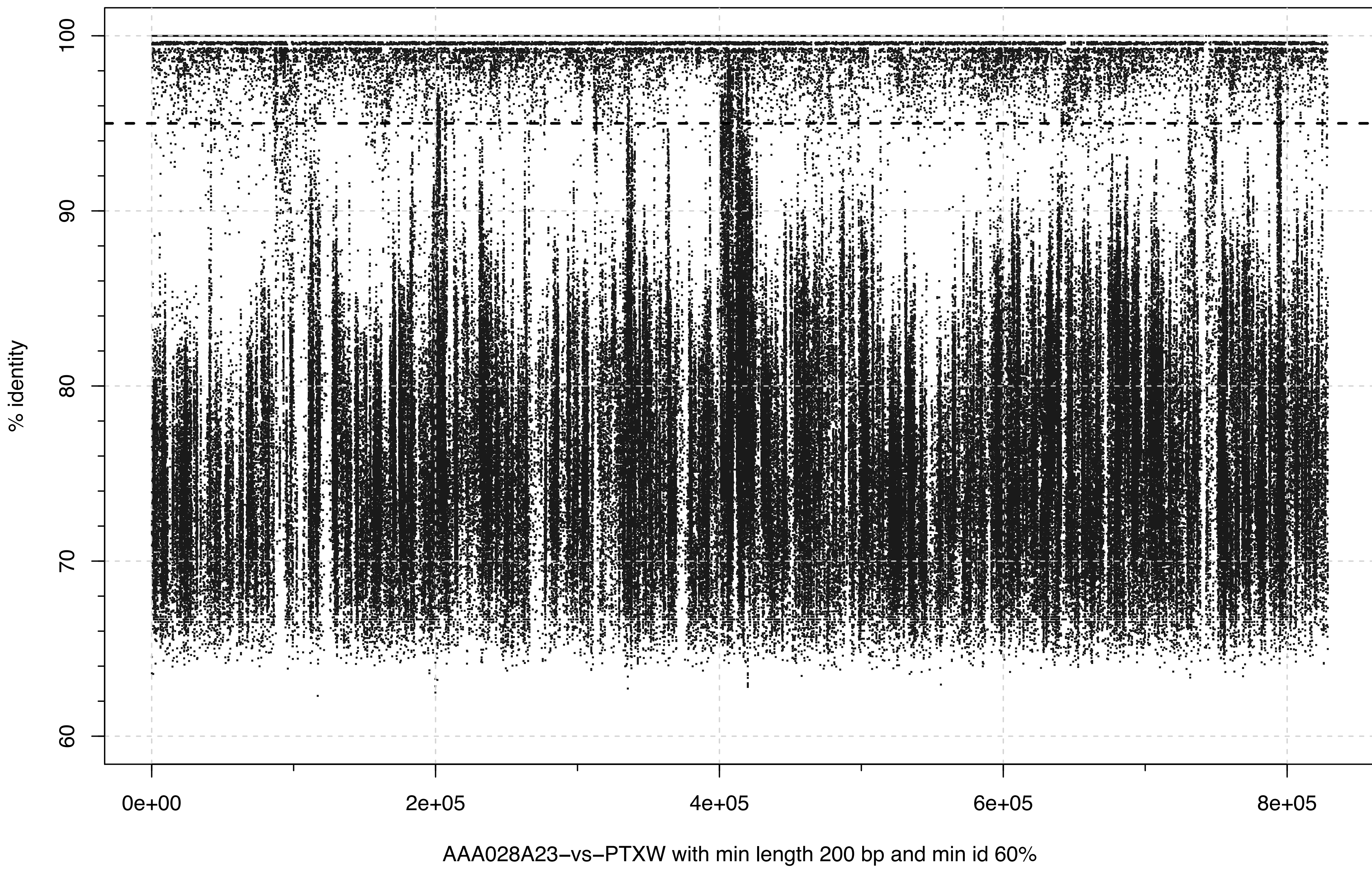

100
90
80
70
60
% identity
0e+00
2e+05
4e+05
6e+05
8e+05
AAA028A23−vs−PTXW with min length 200 bp and min id 60%

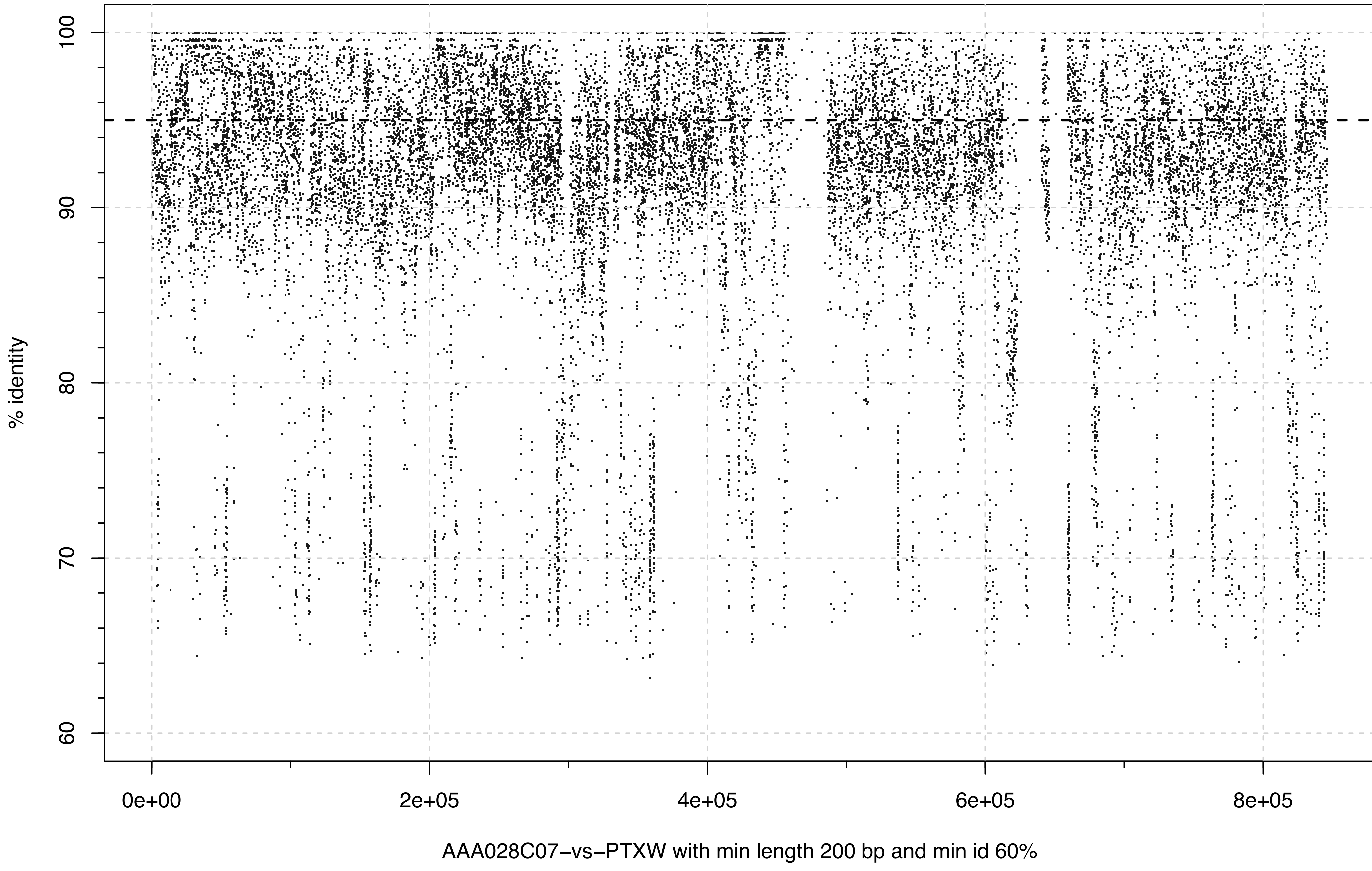

100
90
80
70
60
% identity
0e+00
2e+05
4e+05
6e+05
8e+05
AAA028C07−vs−PTXW with min length 200 bp and min id 60%

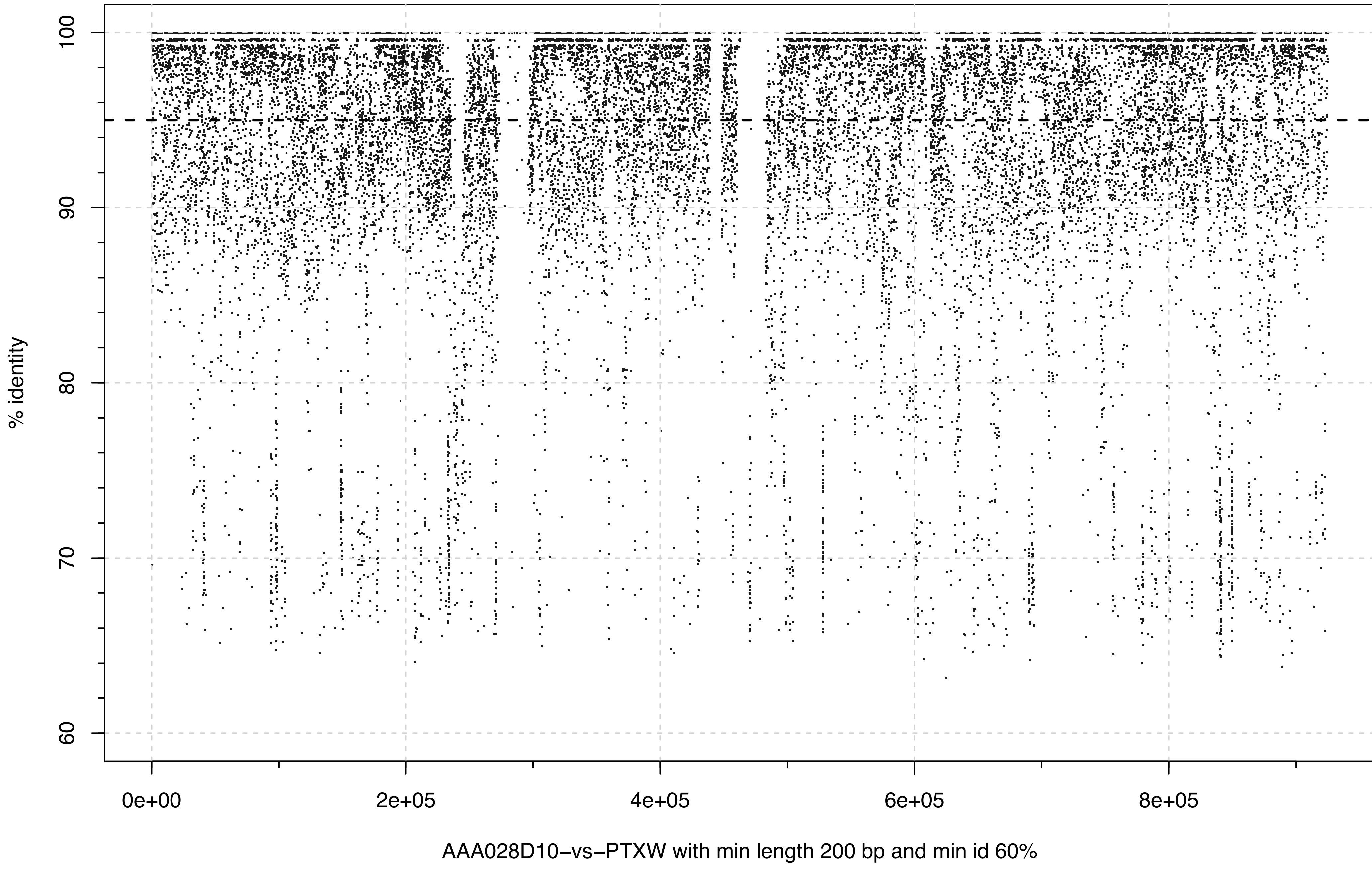

100
90
80
70
60
% identity
0e+00
2e+05
4e+05
6e+05
8e+05
AAA028D10−vs−PTXW with min length 200 bp and min id 60%

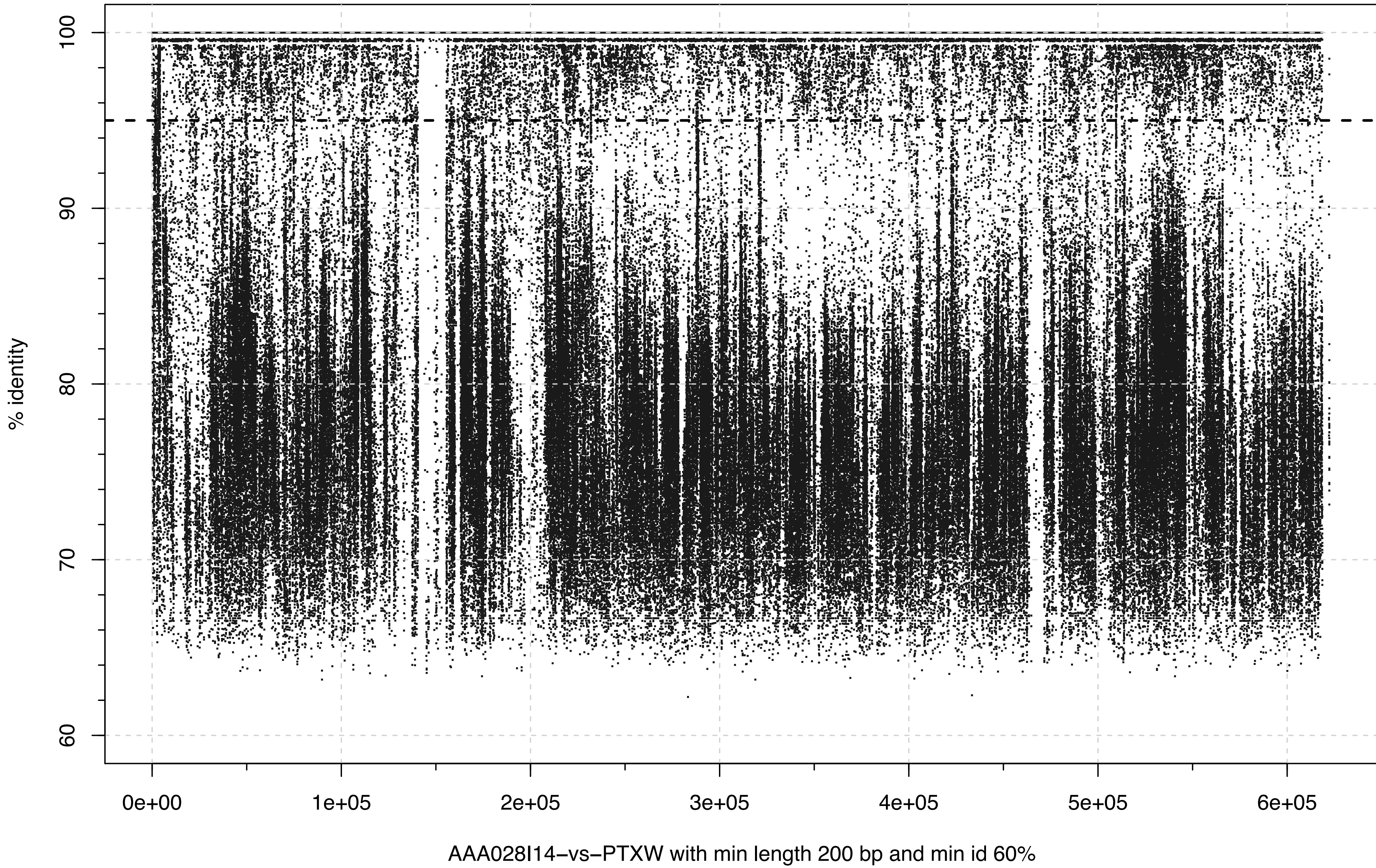

% identity
100
90
80
70
60
0e+00
1e+05
2e+05
3e+05
4e+05
5e+05
6e+05
AAA028I14−vs−PTXW with min length 200 bp and min id 60%

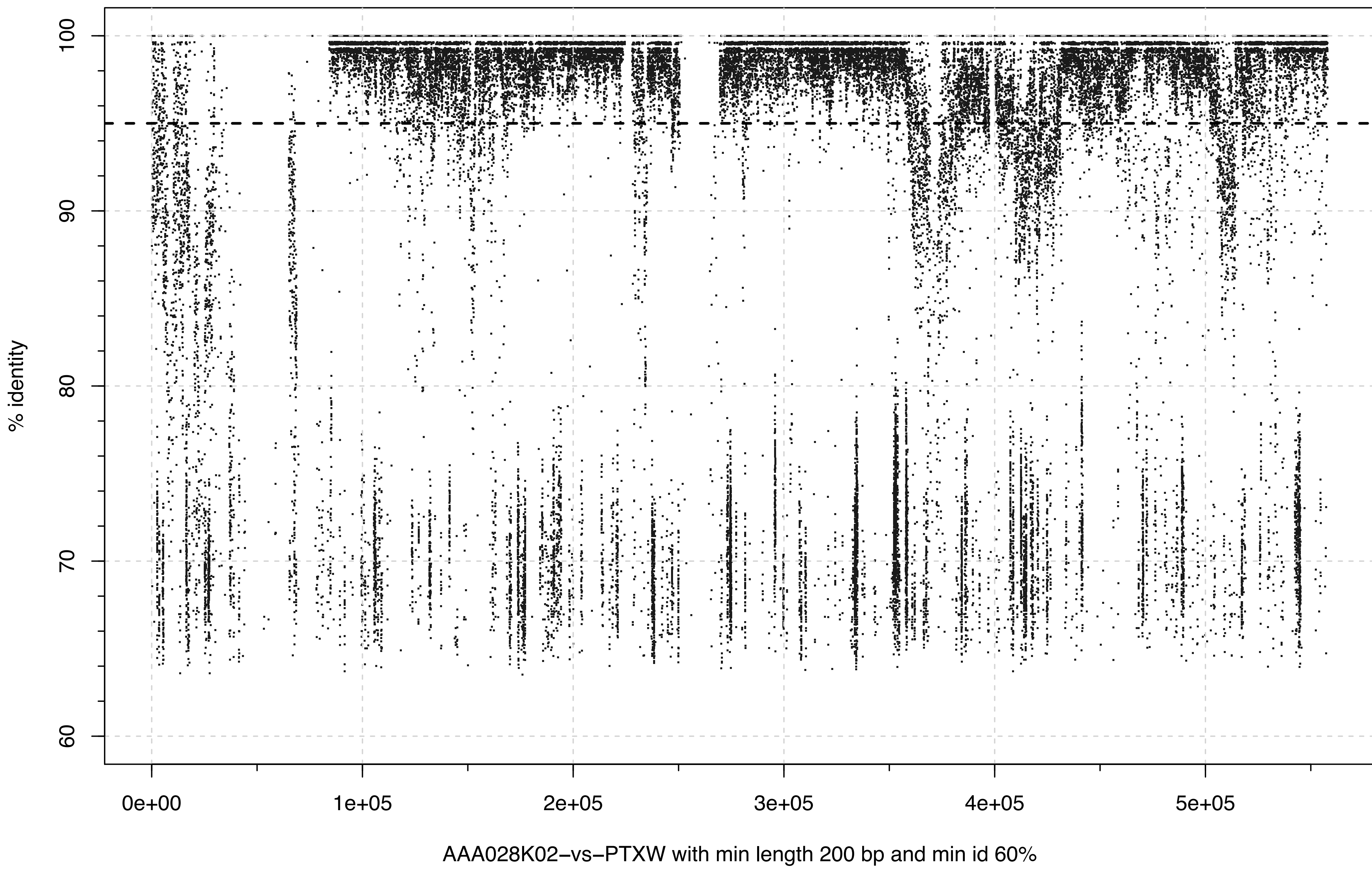

% identity
100
90
80
70
60
0e+00
1e+05
2e+05
3e+05
4e+05
5e+05
AAA028K02−vs−PTXW with min length 200 bp and min id 60%

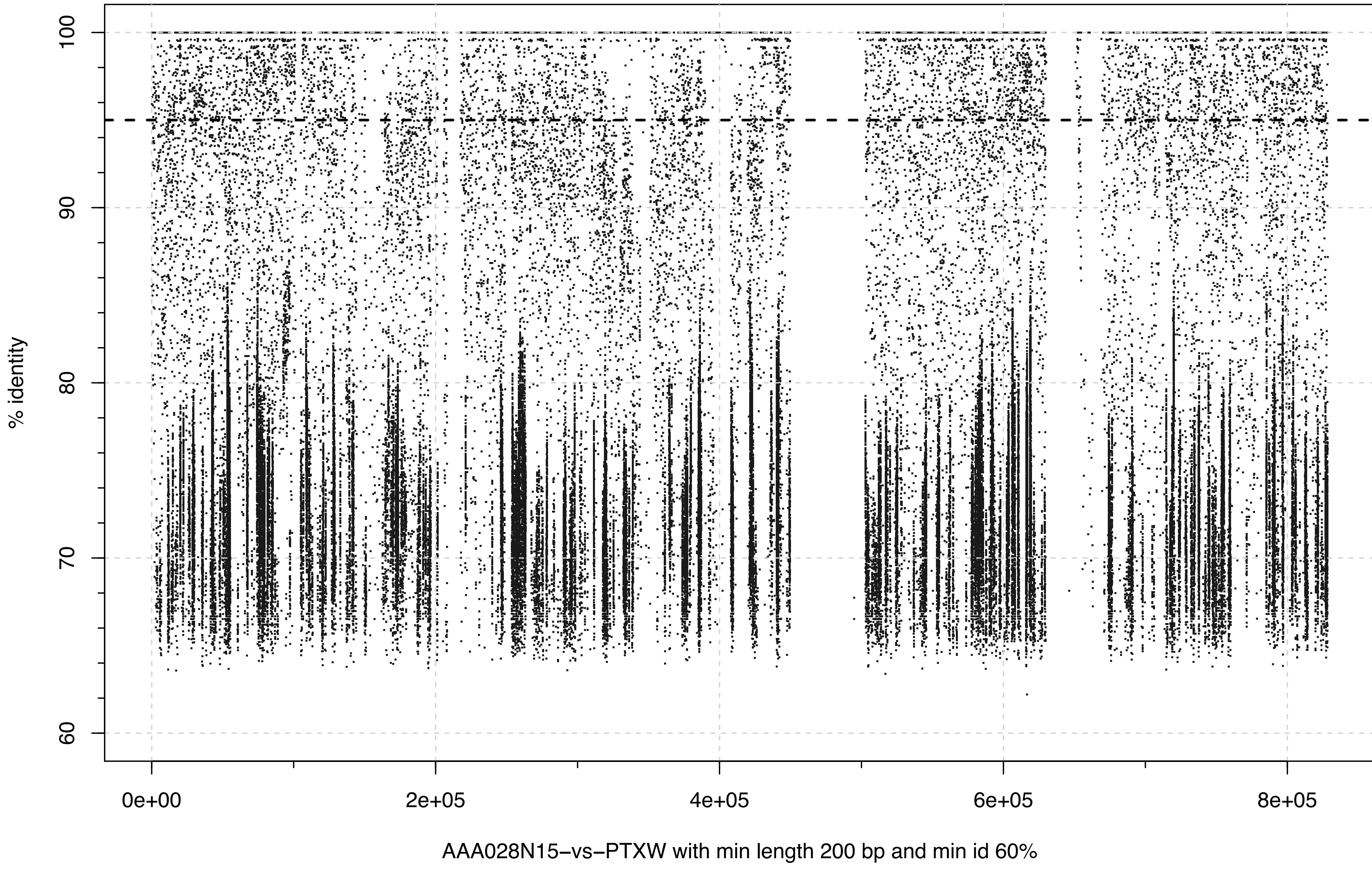

% identity
100
90
80
70
60
0e+00
2e+05
4e+05
6e+05
8e+05
AAA028N15−vs−PTXW with min length 200 bp and min id 60%

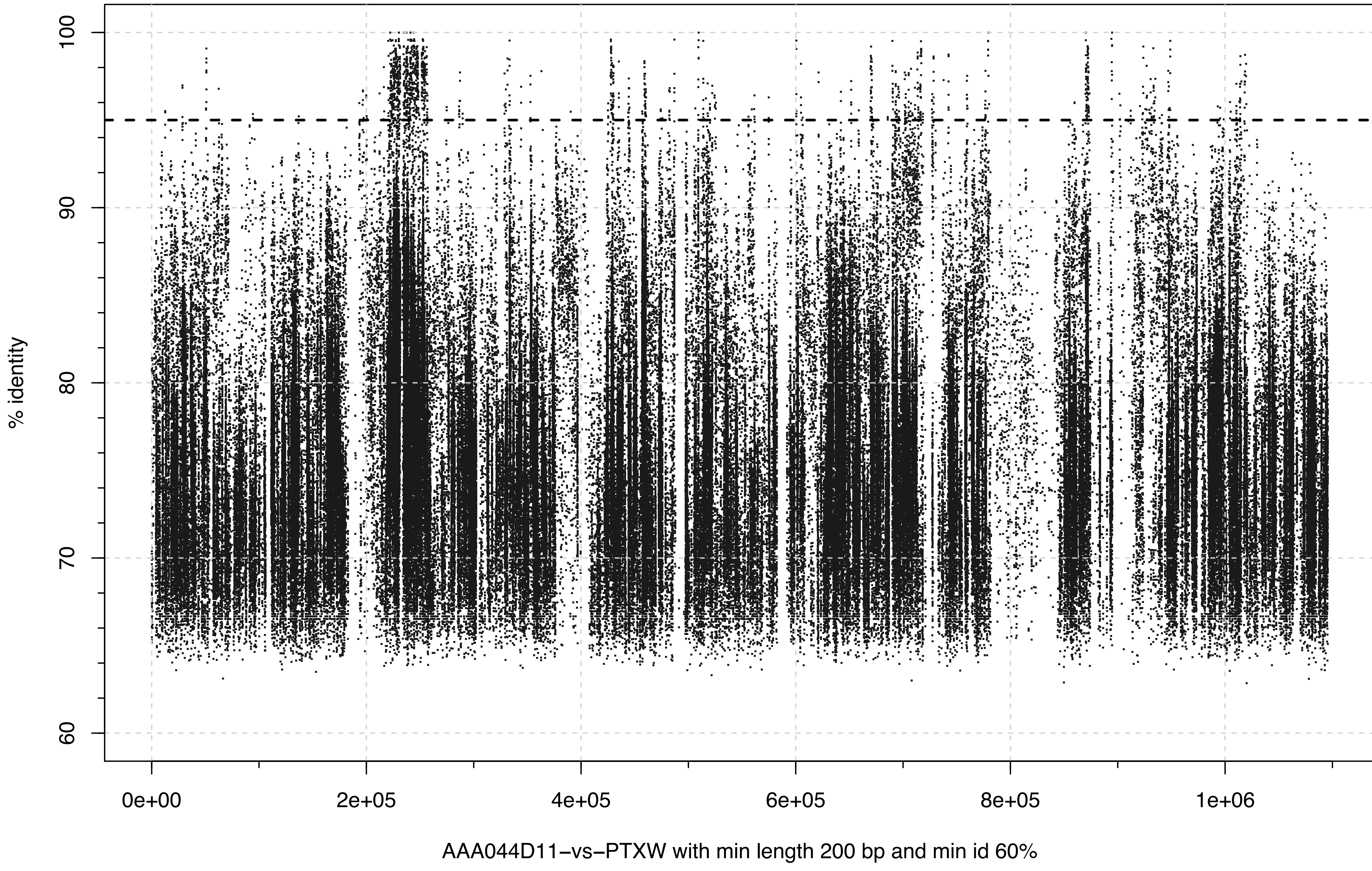

100
90
80
70
60
% identity
0e+00
2e+05
4e+05
6e+05
8e+05
1e+06
AAA044D11−vs−PTXW with min length 200 bp and min id 60%

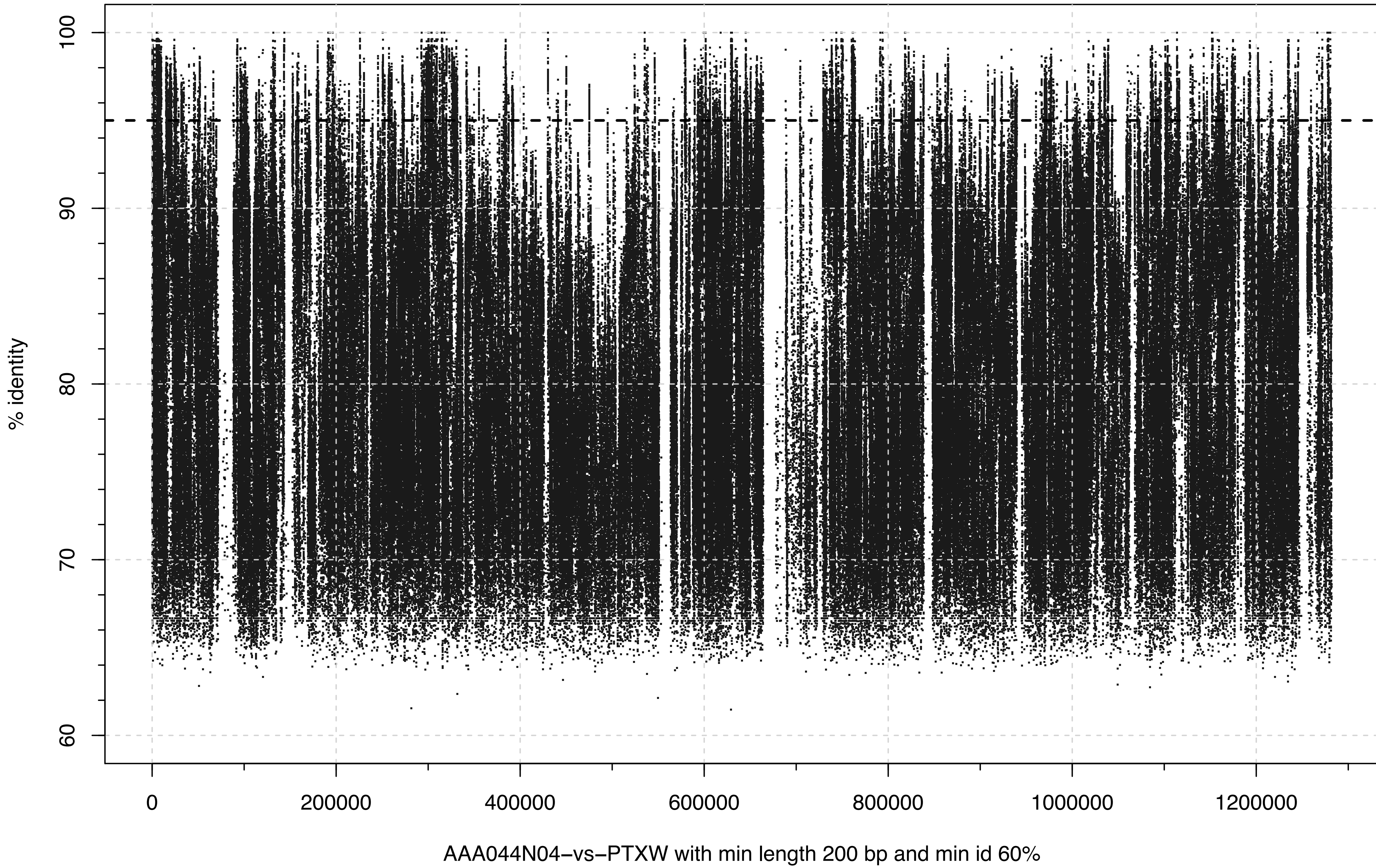

100
90
80
70
60
% identity
0
200000
400000
600000
800000
1000000
1200000
AAA044N04−vs−PTXW with min length 200 bp and min id 60%

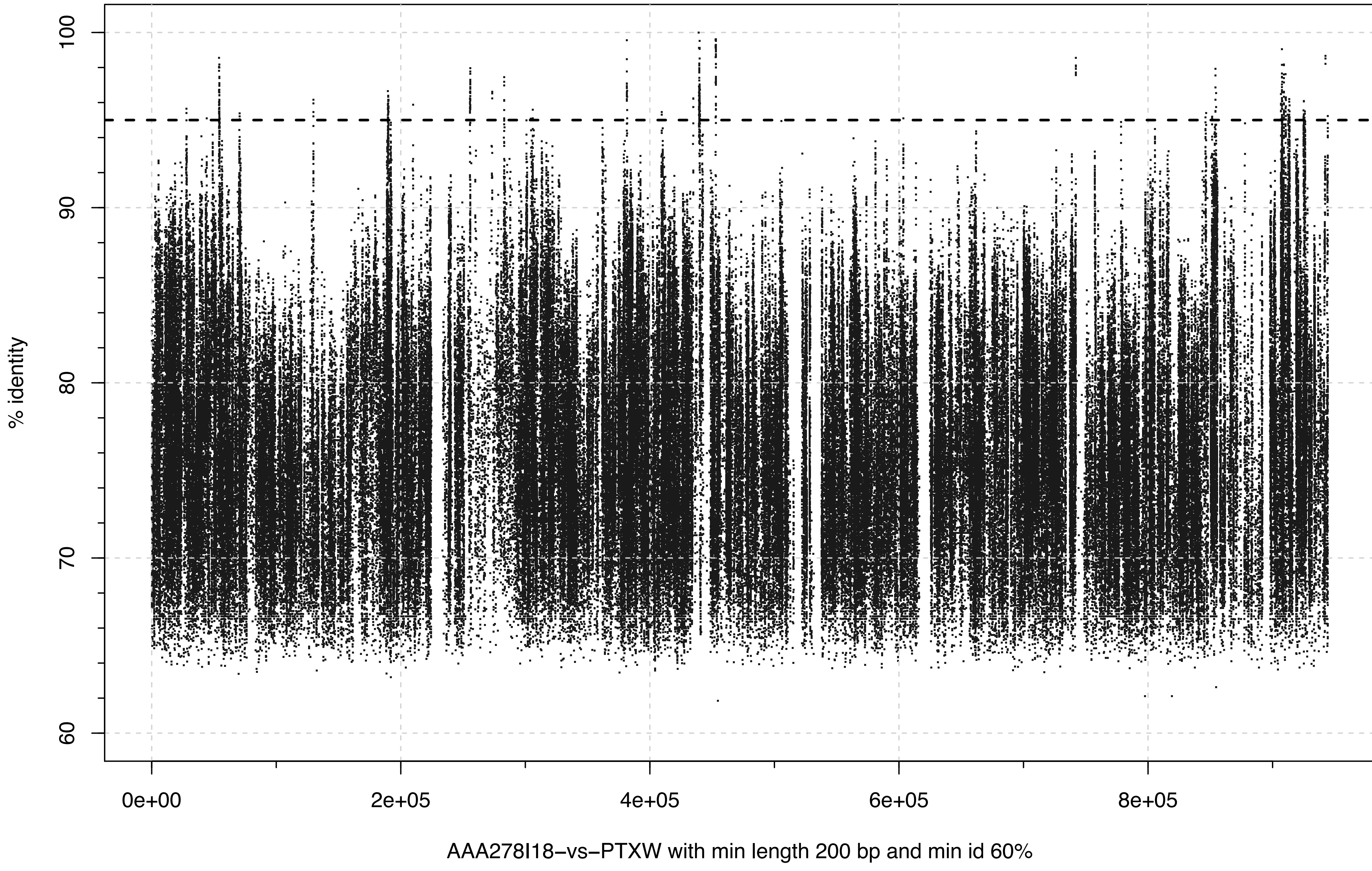

100
90
80
70
60
% identity
0e+00
2e+05
4e+05
6e+05
8e+05
AAA278I18−vs−PTXW with min length 200 bp and min id 60%

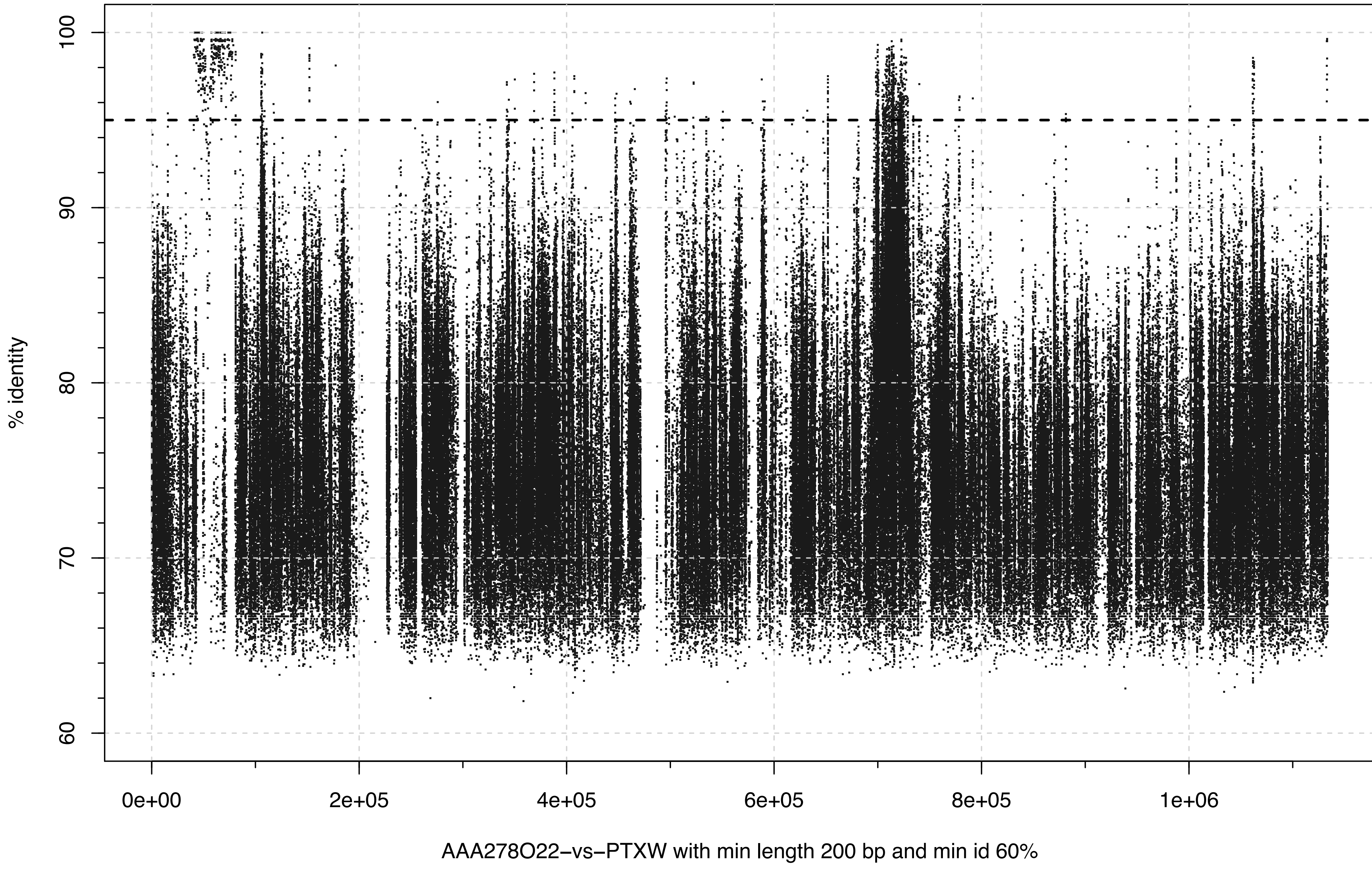
% identity
100
90
80
70
60
0e+00
2e+05
4e+05
6e+05
8e+05
1e+06
AAA278O22−vs−PTXW with min length 200 bp and min id 60%

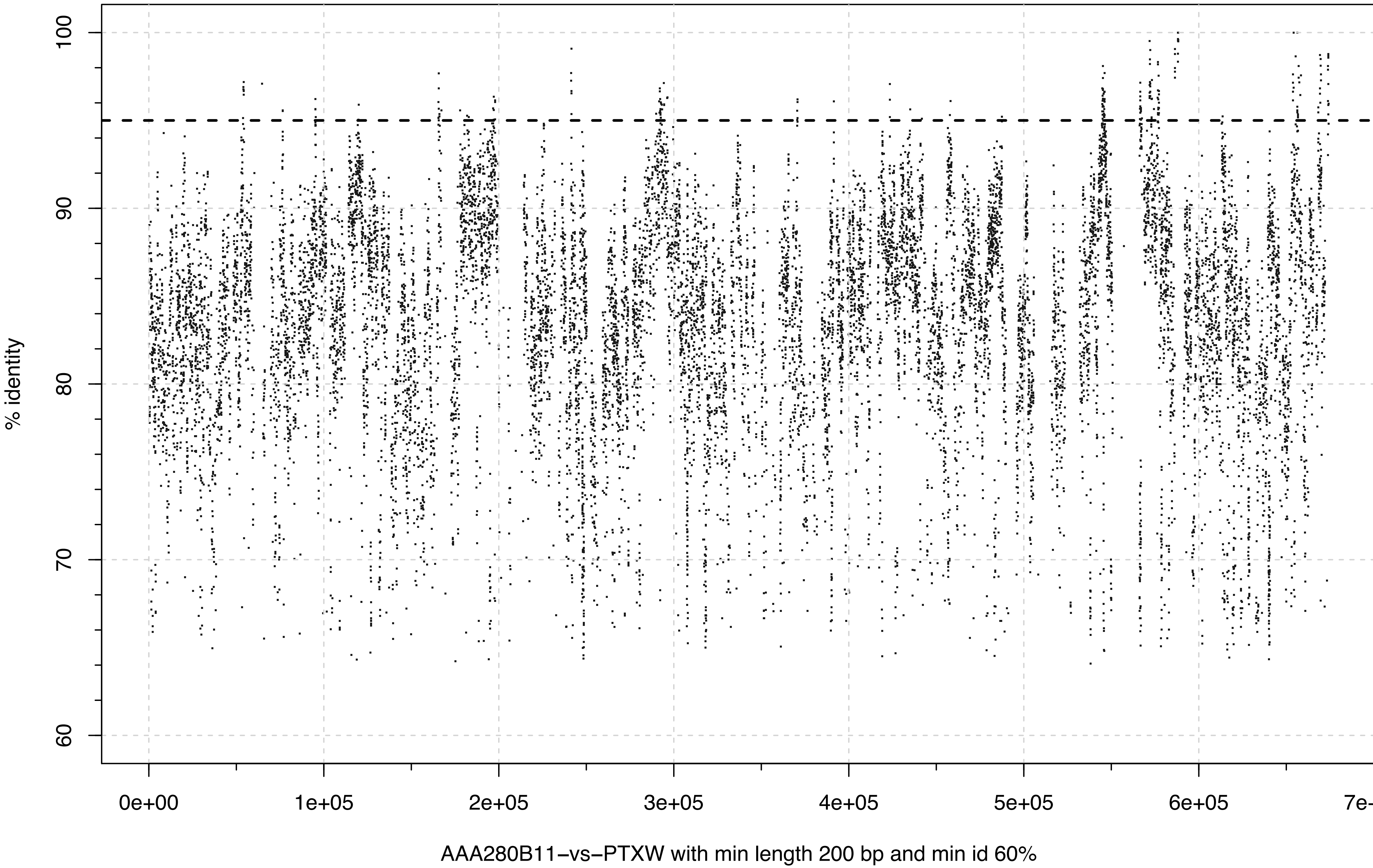
% identity
100
90
80
70
60
0e+00
1e+05
2e+05
3e+05
4e+05
5e+05
6e+05
AAA280B11−vs−PTXW with min length 200 bp and min id 60%

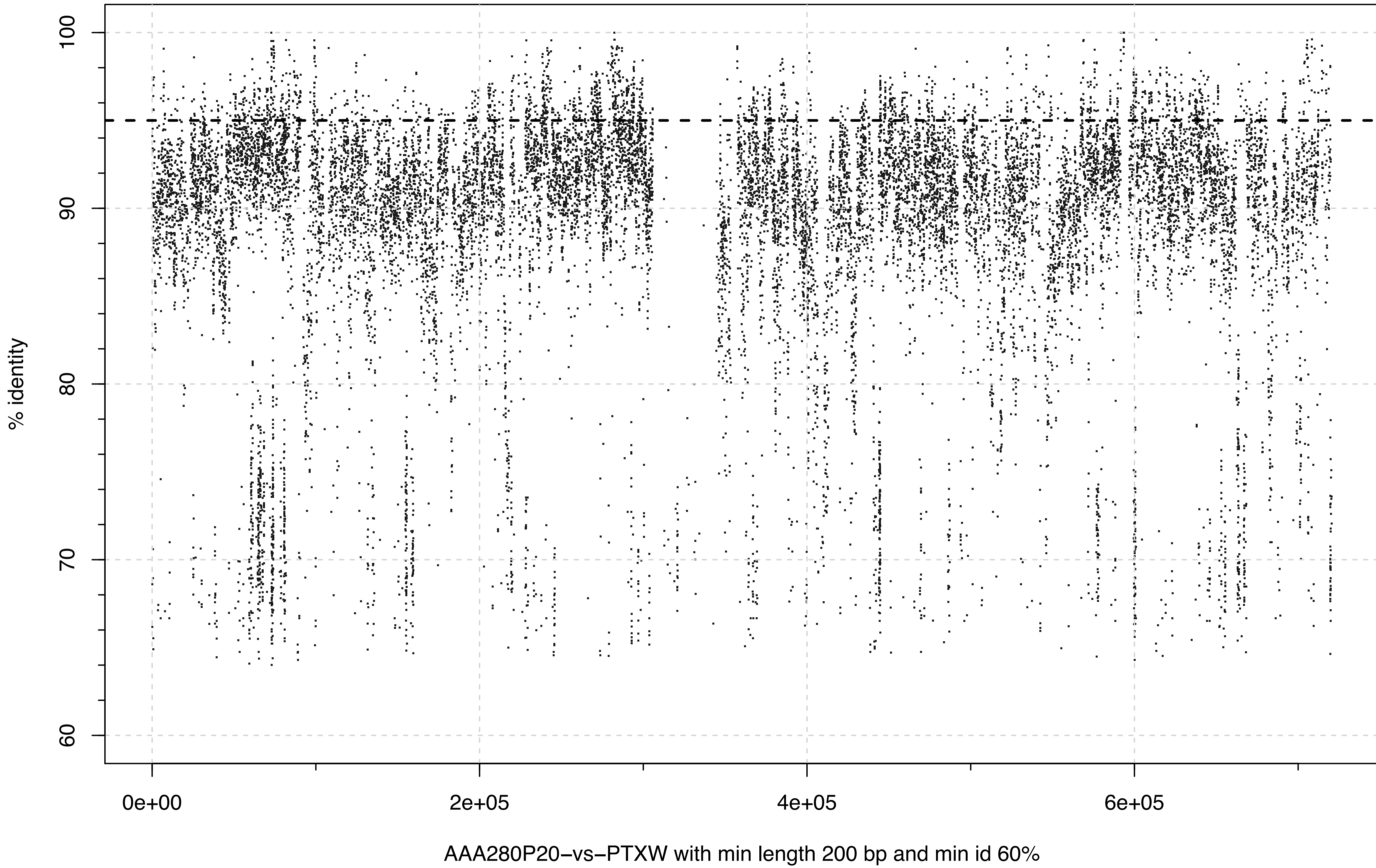

100
90
80
70
60
% identity
0e+00
2e+05
4e+05
6e+05
AAA280P20−vs−PTXW with min length 200 bp and min id 60%

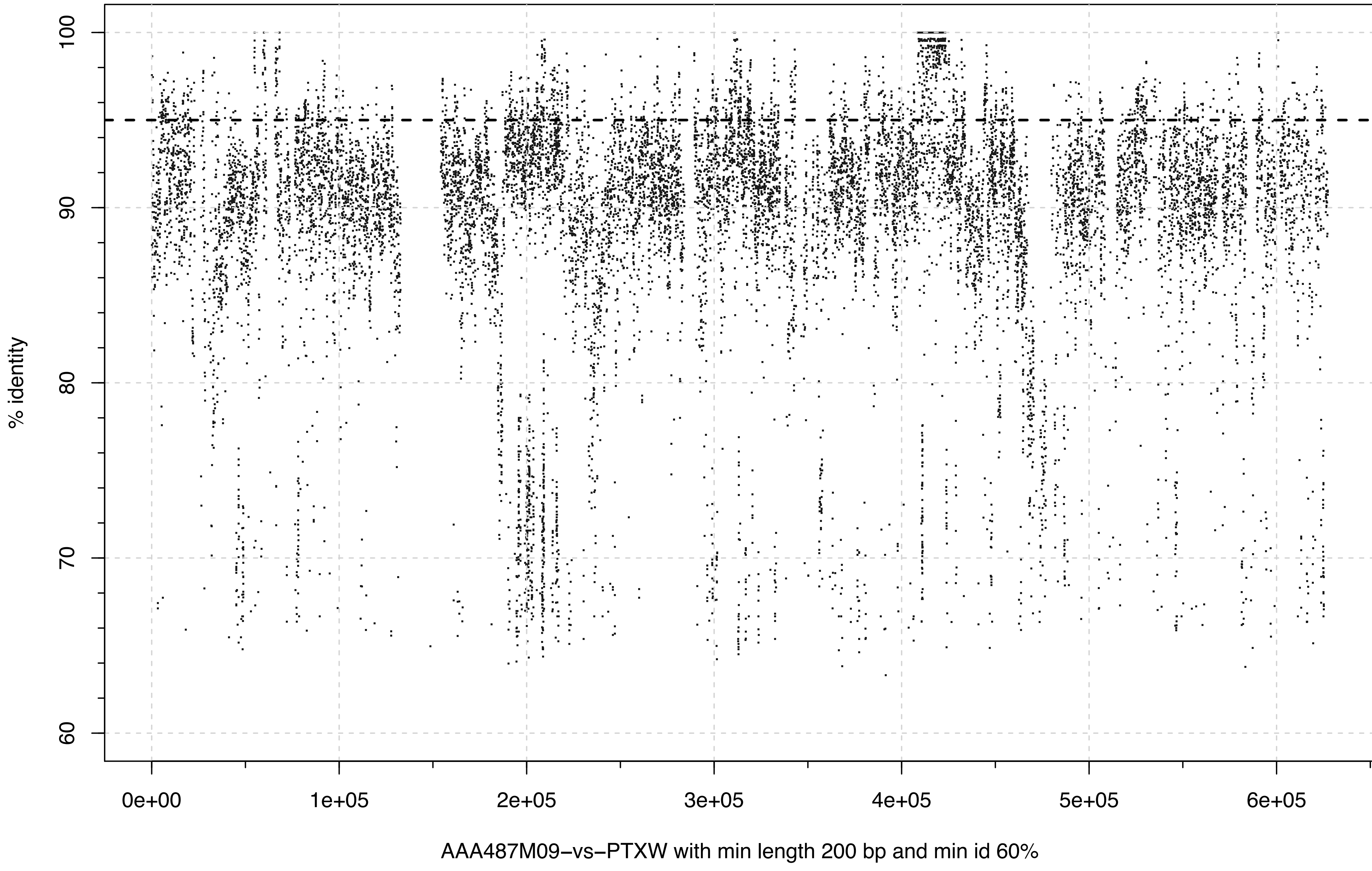

% identity
100
90
80
70
60
0e+00
1e+05
2e+05
3e+05
4e+05
5e+05
6e+05
AAA487M09−vs−PTXW with min length 200 bp and min id 60%

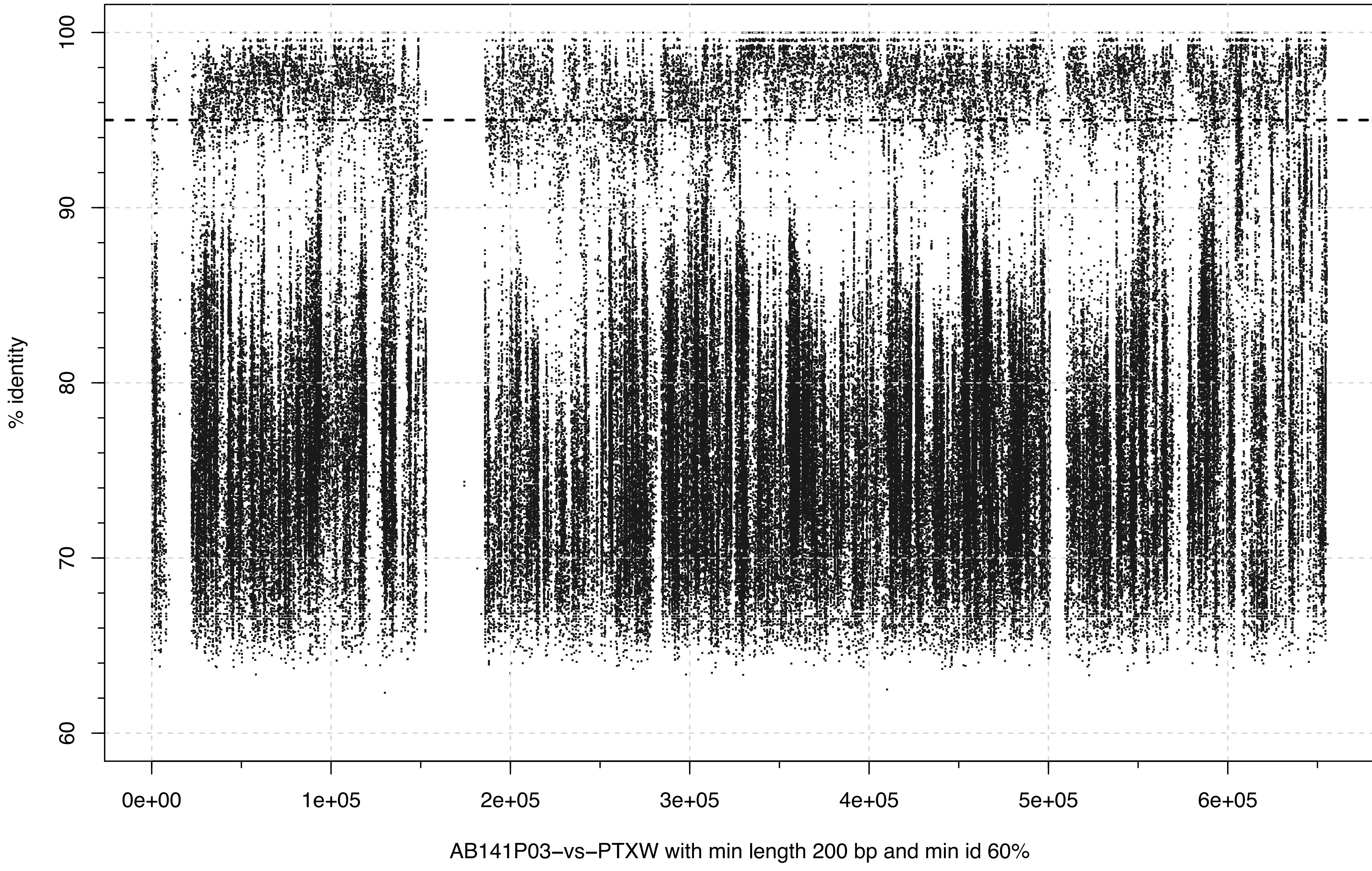

% identity
100
90
80
70
60
0e+00
1e+05
2e+05
3e+05
4e+05
5e+05
6e+05
AB141P03−vs−PTXW with min length 200 bp and min id 60%
