# Supplementary figures and images for: Contrasting patterns of genome-level diversity across distinct co-occurring bacterial populations
Source: ISME J. 2017 Dec 8;12(3):742–55. doi: 10.1038/s41396-017-0001-0 (PMC5962901; doi:10.1038/s41396-017-0001-0)

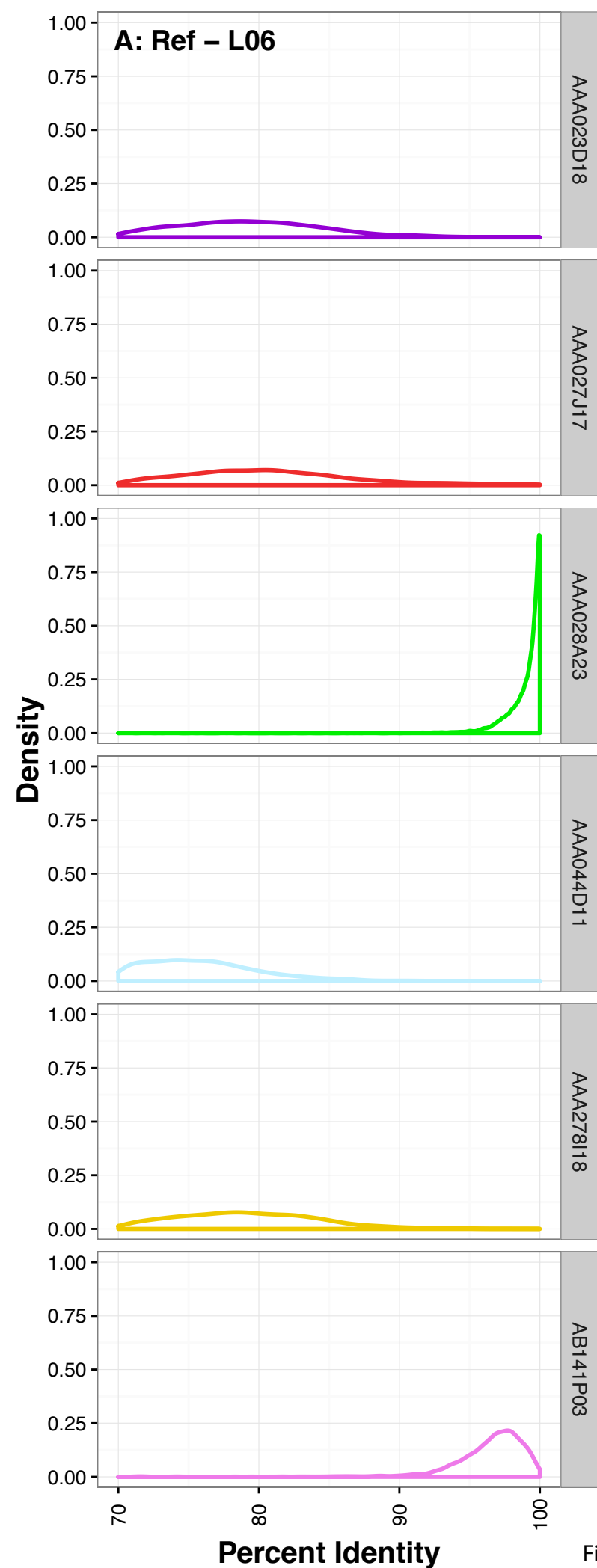

Figure S1

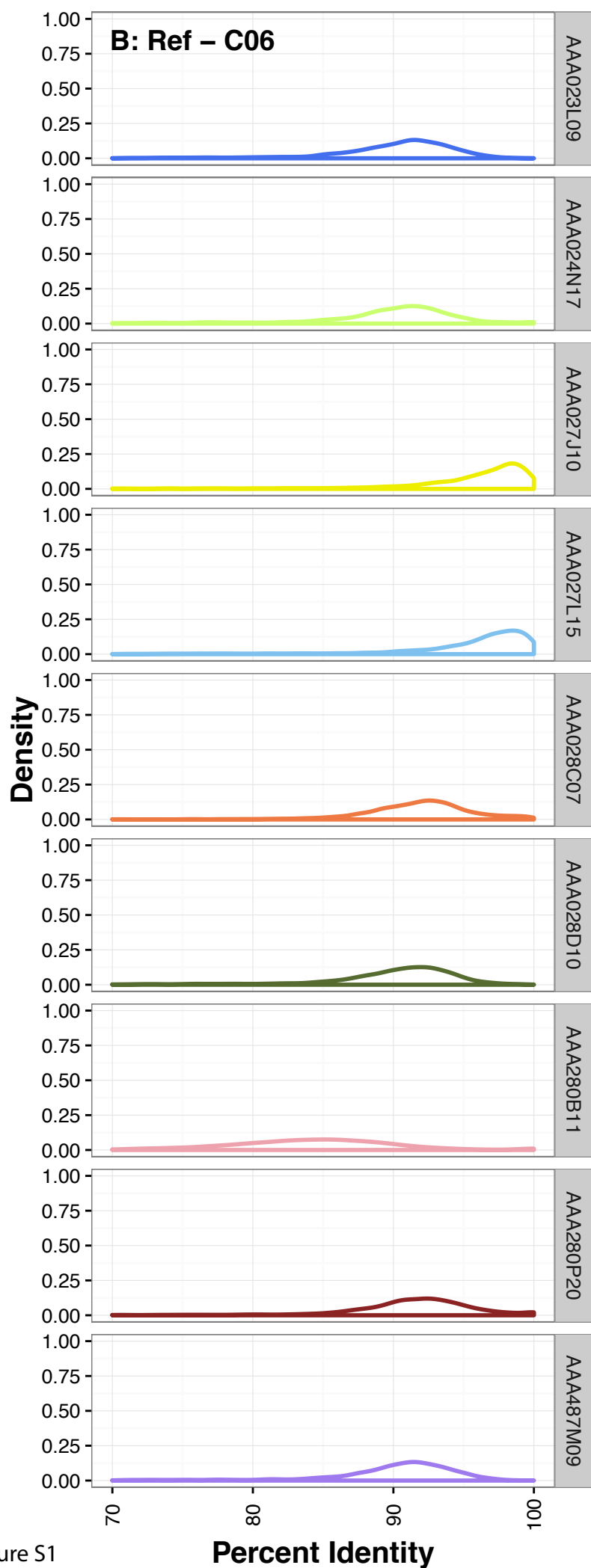

Supplement: Supplementary file 3 — Supplemental Figures [file 41396_2017_1_MOESM3_ESM.zip › FigS1-SAG-vs-SAG_facet_den_plots.pdf]

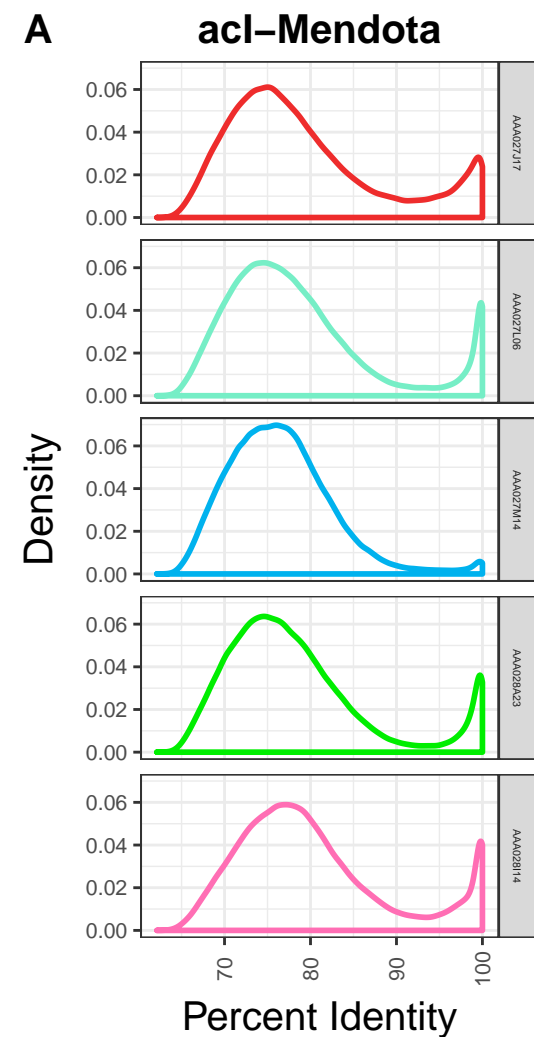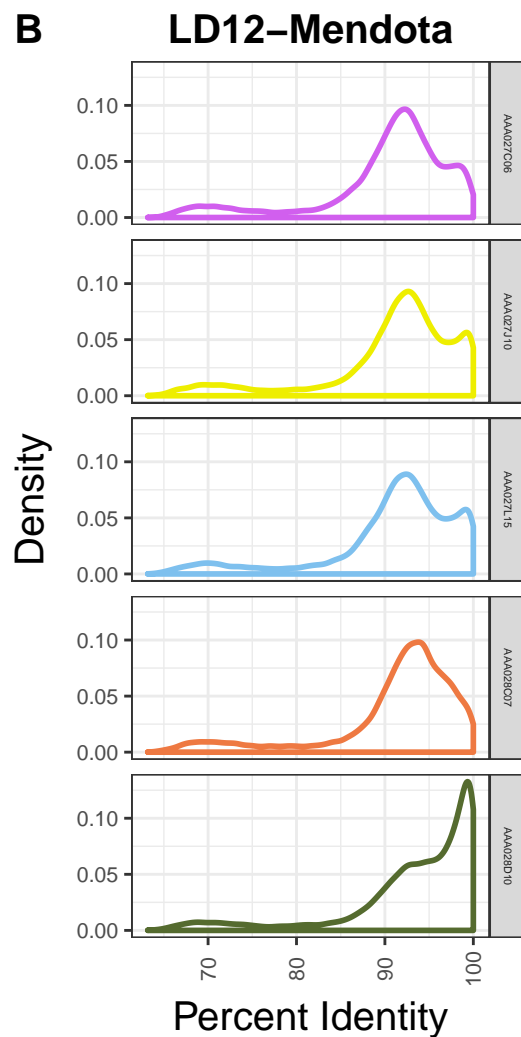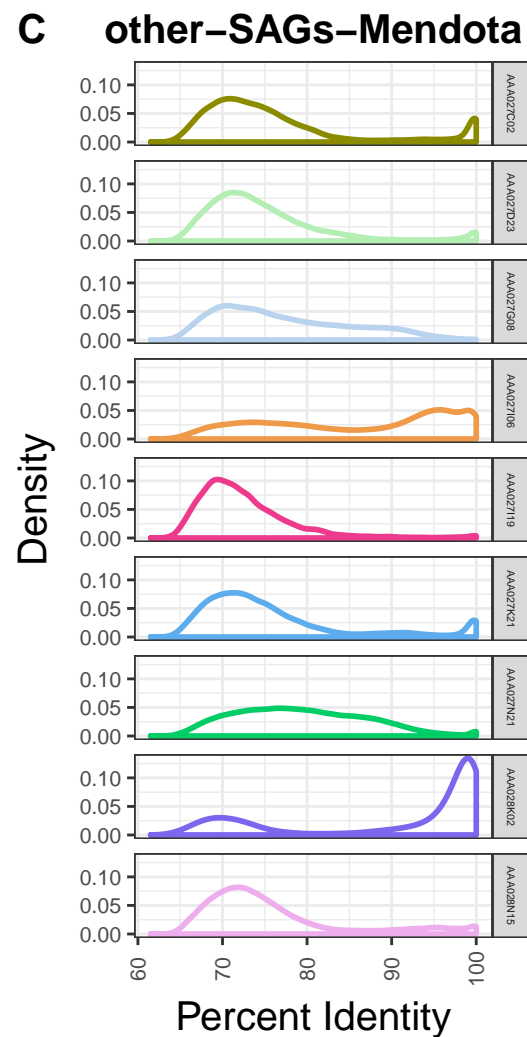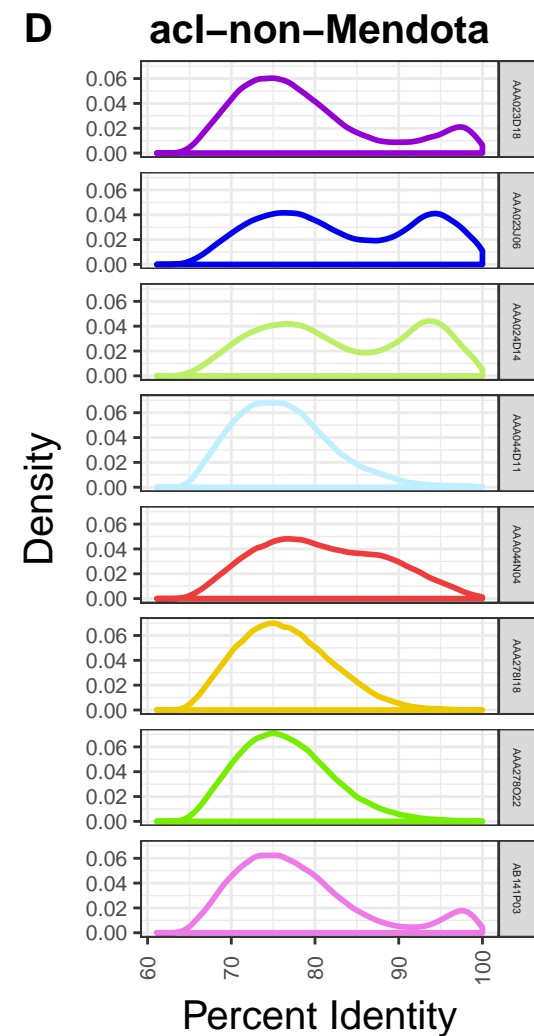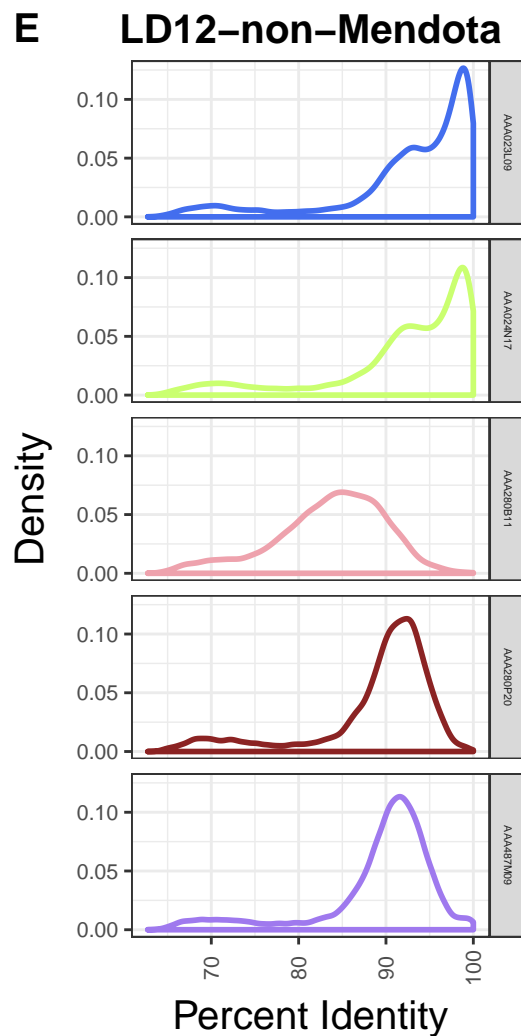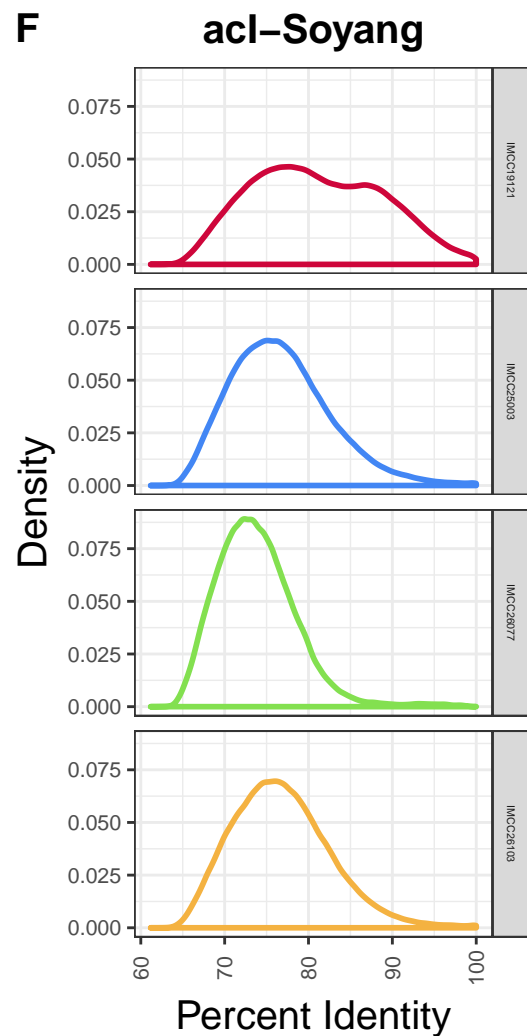

Supplement: Supplementary file 3 — Supplemental Figures [file 41396_2017_1_MOESM3_ESM.zip › FigS3-facet_seqdiscplots.pdf]

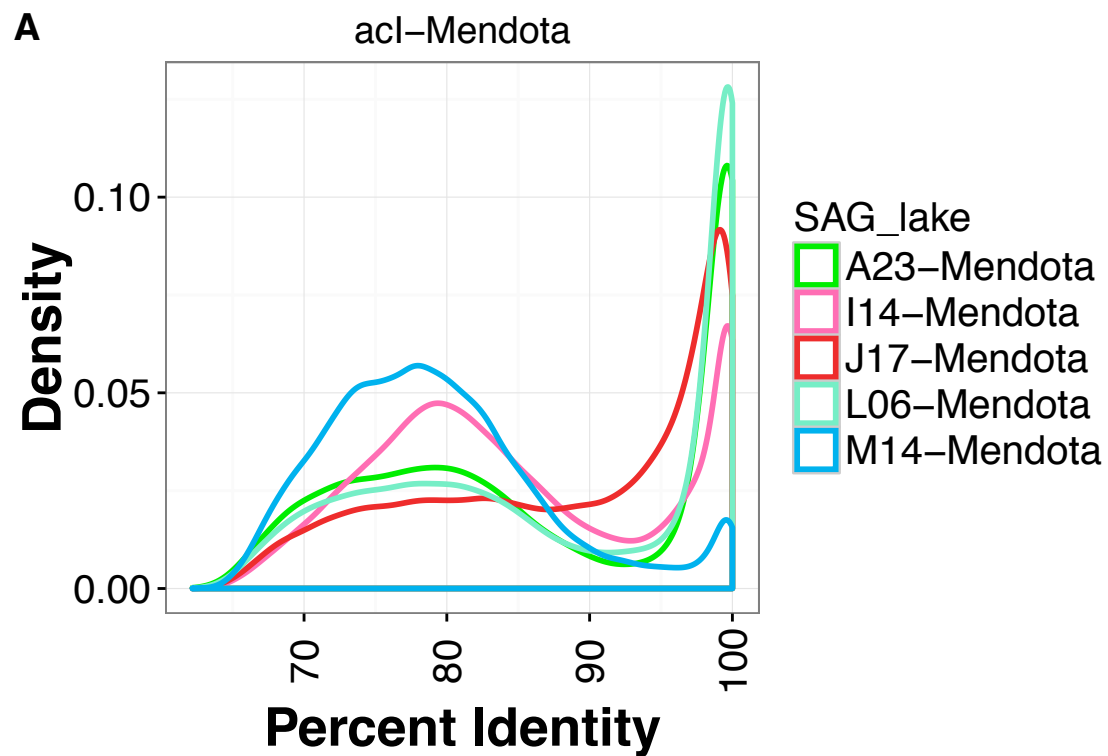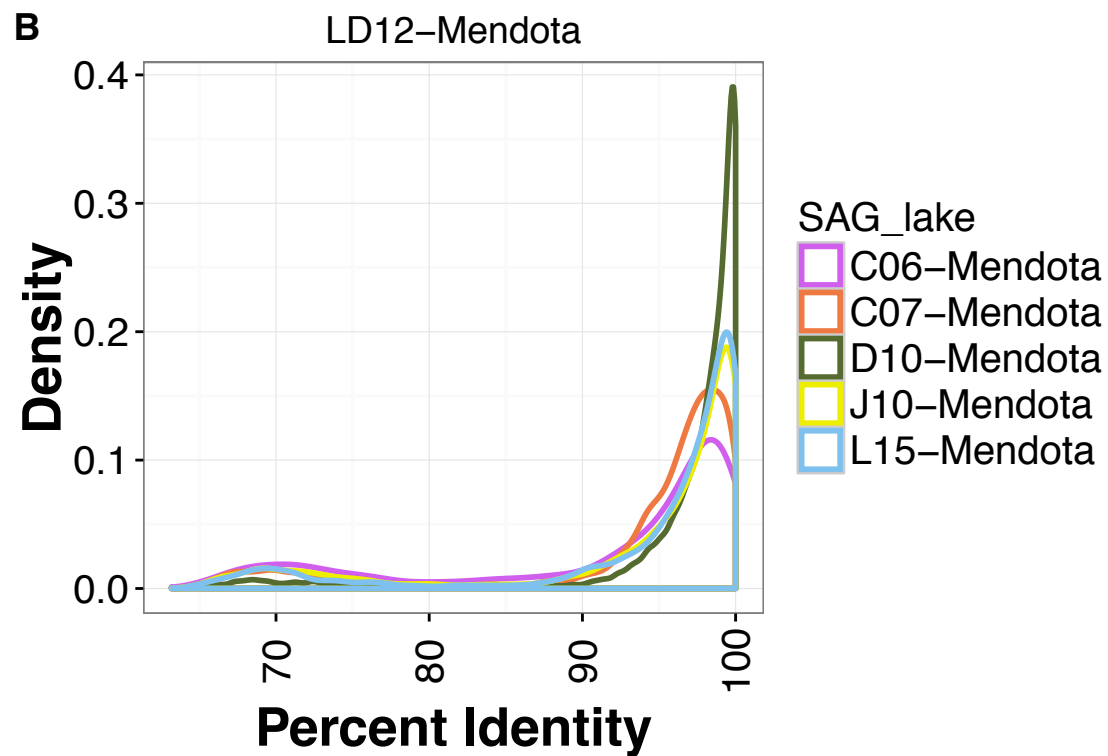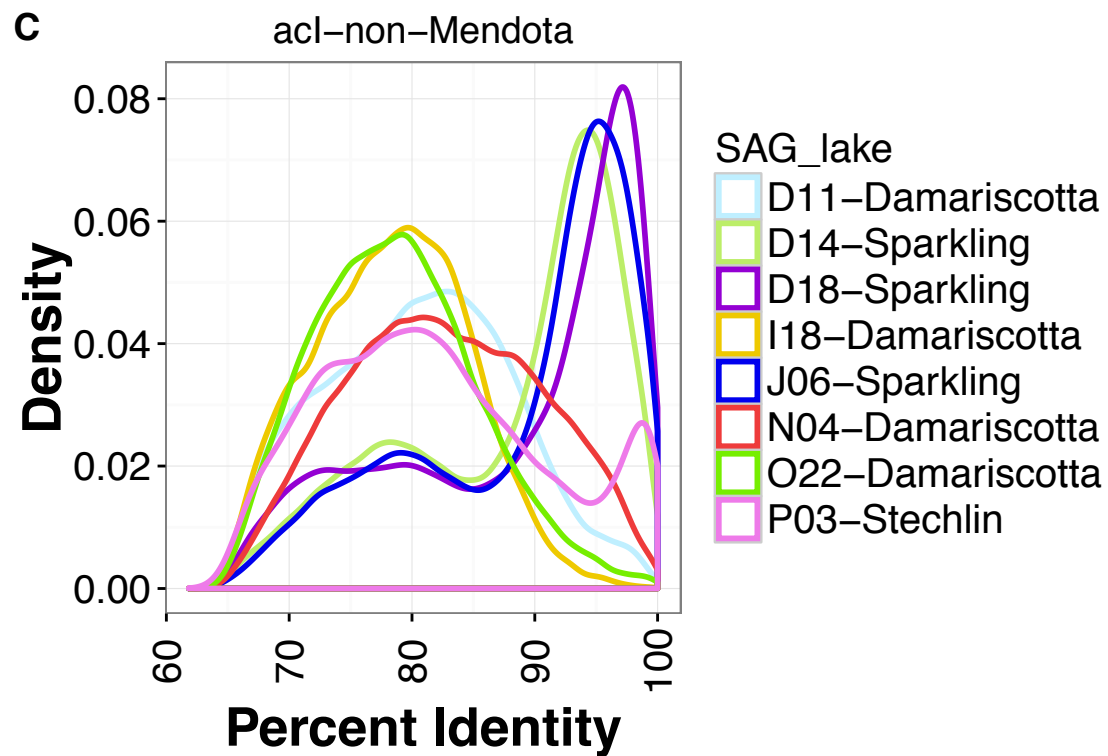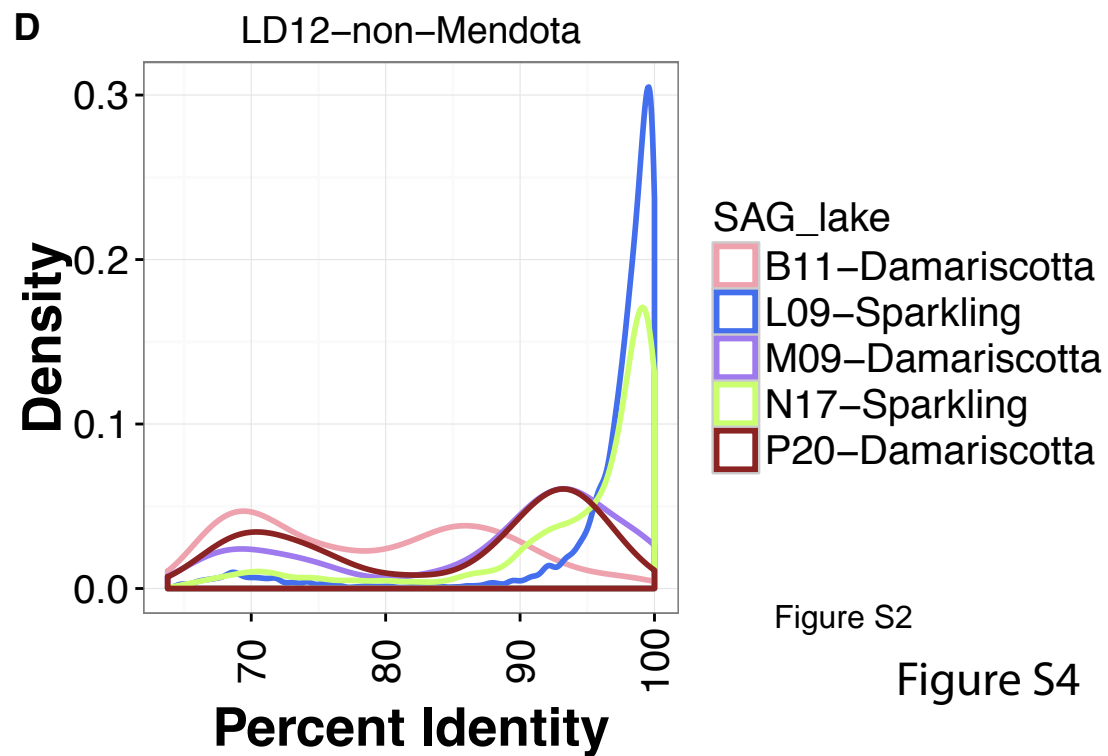

Figure S2

Figure S4

Supplement: Supplementary file 3 — Supplemental Figures [file 41396_2017_1_MOESM3_ESM.zip › FigS4-competitive-seqdiscdenplots-20161004.pdf]

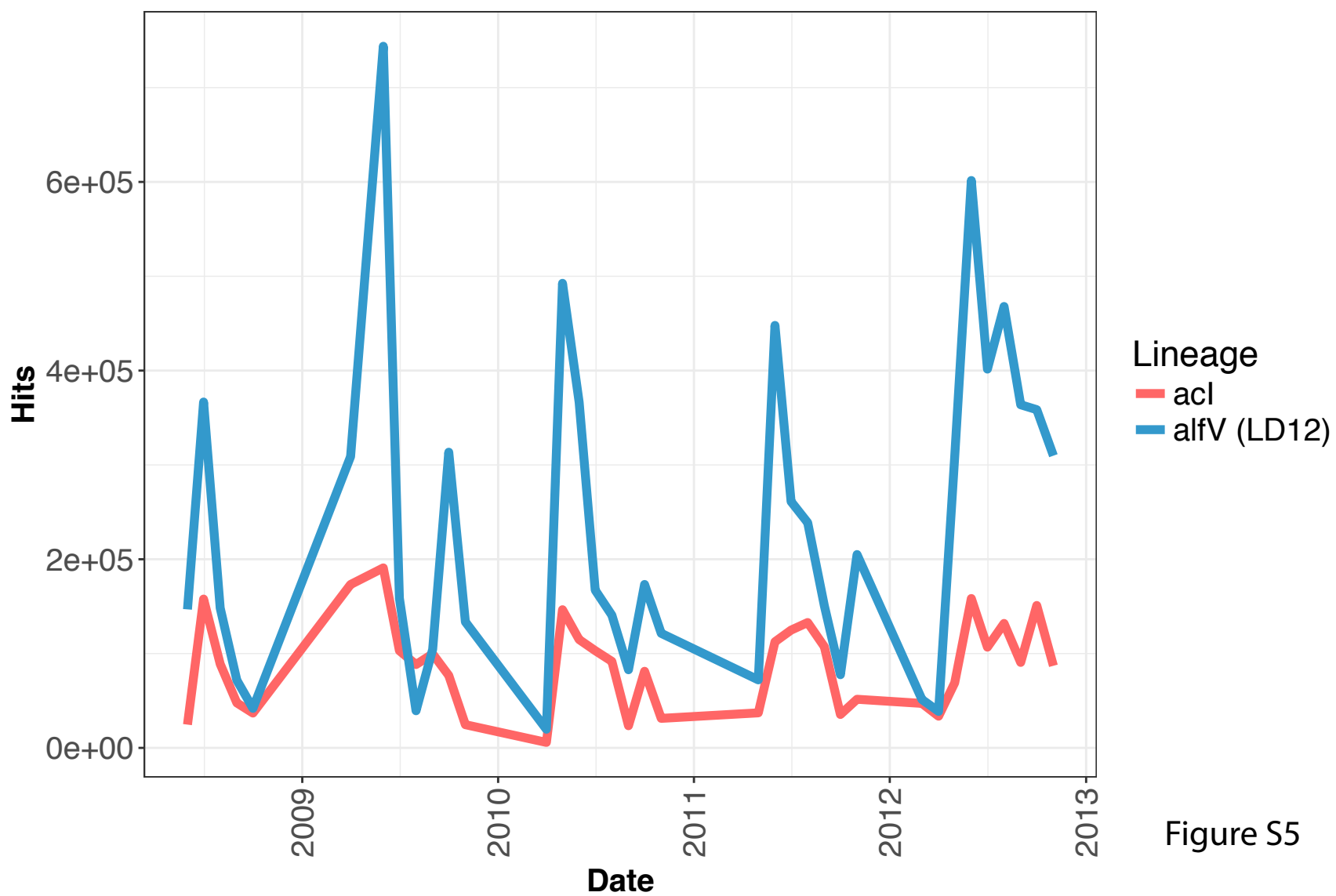

Figure S5

Supplement: Supplementary file 3 — Supplemental Figures [file 41396_2017_1_MOESM3_ESM.zip › FigS5-hitplot-LD12-acI.pdf]

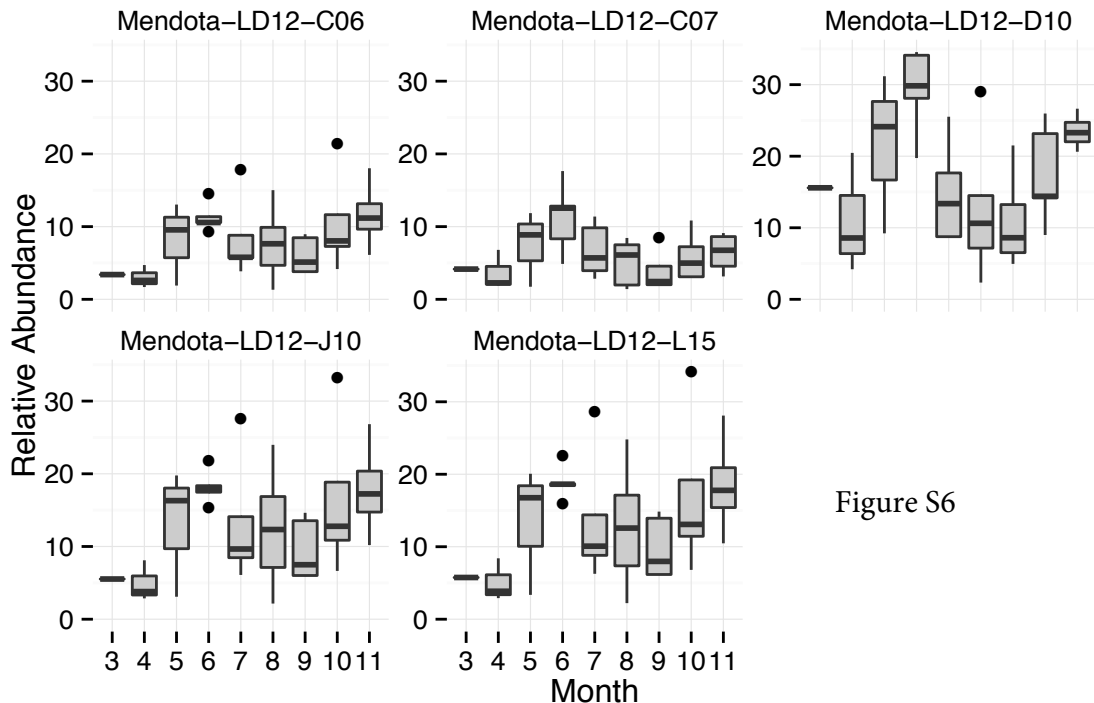

Figure S6

Supplement: Supplementary file 3 — Supplemental Figures [file 41396_2017_1_MOESM3_ESM.zip › FigS6-AbundanceVariance.filtered.LD12-20160408.pdf]

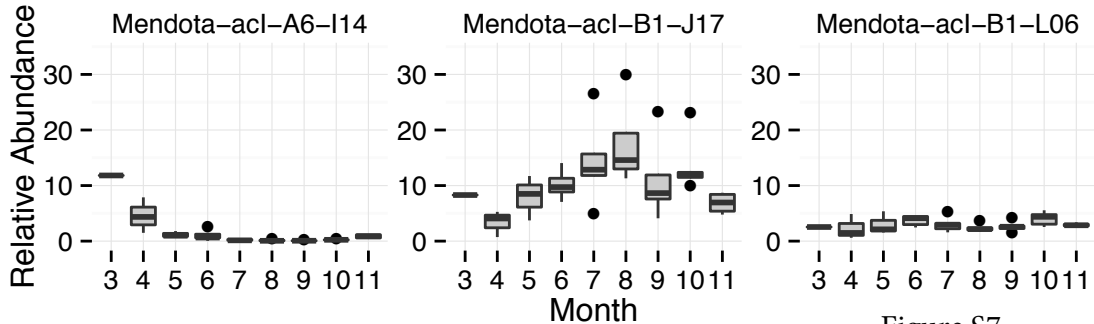

Figure S7

Supplement: Supplementary file 3 — Supplemental Figures [file 41396_2017_1_MOESM3_ESM.zip › FigS7-AbundanceVariance.filtered.acI-20160408.pdf]

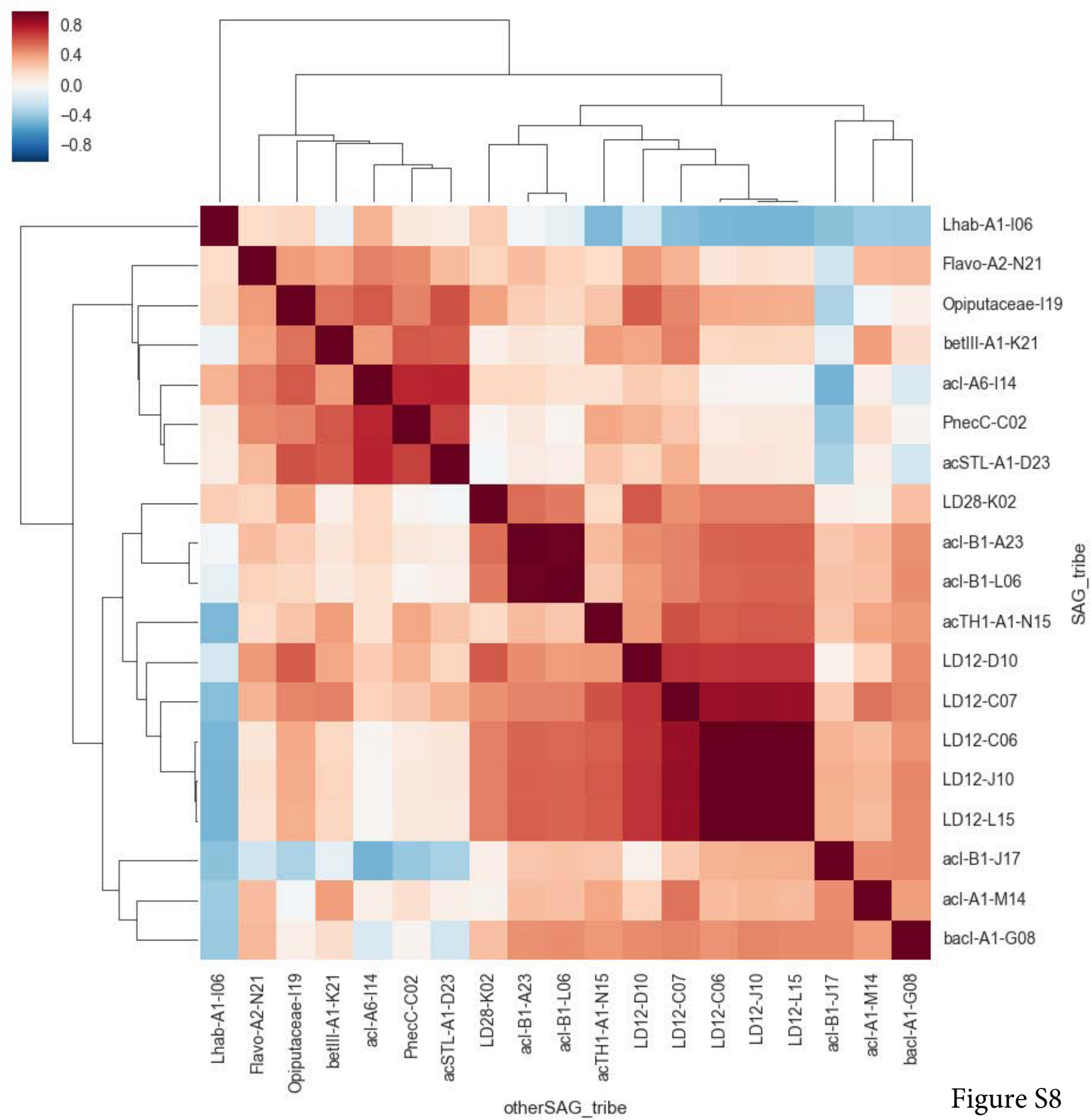

Figure S8

Supplement: Supplementary file 3 — Supplemental Figures [file 41396_2017_1_MOESM3_ESM.zip › FigS8-MEcorrelHeatmap-20161101.pdf]
